# The Effects of Forecasts on the Accuracy and Precision of Expectations

**DOI:** 10.1093/poq/nfaf003

**Published:** 2025-05-09

**Authors:** Matthew Barnfield, Joseph Phillips, Florian Stoeckel, Benjamin Lyons, Paula Szewach, Jack Thompson, Vittorio Mérola, Sabrina Stöckli, Jason Reifler

**Affiliations:** Postdoctoral Fellow, School of Politics and International Relations, Queen Mary University of London, London, UK; Lecturer, School of Law and Politics, Cardiff University, Cardiff, UK; Associate Professor, Department of Politics, University of Exeter, Exeter, UK; Assistant Professor, Department of Communication, University of Utah, Salt Lake City, UT, US; Recognized Researcher, Barcelona Supercomputing Centre, Barcelona, Spain; Postdoctoral Research Fellow, Leeds University Business School, University of Leeds, Leeds, UK; Assistant Professor, School of Government and International Affairs, Durham University, Durham, UK; Senior Research Associate, Chair of Marketing, University of Zurich, Zurich, Switzerland; Professor, Department of Politics and International Relations, University of Southampton, Southampton, UK

## Abstract

Quantitative forecasts have become increasingly prominent as tools for aiding public understanding of sociopolitical trends. But how much, and what, do people learn from quantitative forecasts? In this note, we show through a preregistered survey experiment that real forecasts of the 2022 French presidential election significantly affect expectations of the election result. The direction of that effect hinges on how the forecast is presented. Voters become more accurate and precise in their predictions of each candidate’s vote share when given forecast information in the form of projected vote share. Forecasts presented as numerical probabilities make such expectations *less* accurate and *less* precise. When combined, the effects of both forms on vote share expectations tend to cancel out, but jointly boost voters’ ability to identify likely winners. Our findings have implications for the public communication of quantitative information.

## Introduction

Quantitative forecasts of future events have become a cornerstone of media coverage of sociopolitical issues from climate change to COVID-19, and from economics to elections. The growth of such “data journalism” (Pentzold and Fechner 2020) raises the question of whether, and how effectively, forecasts influence public opinion about what the future holds. In this research note, we focus on the case of election forecasts, asking: Do forecasts help or hinder people in forming expectations for the future?

To form such expectations, people respond to currently available information. In the case of election outcomes, voters often rely on vote intention polls (Lavrakas et al. 1991; Irwin and Van Holsteyn 2002; Barnfield 2023b; Stoetzer et al. 2024). Polls, however, are a “snapshot, not a forecast” (Gelman 2013). They convey information on current public opinion, but they cannot straightforwardly be interpreted as projections of the final result. To overcome these limitations, election forecasts supplement aggregated results from a wider pool of polls with historical information and underlying stable factors in an electoral system (Hillygus 2011). It is arguably the primary function of forecasts to help people form credible expectations, rather than to accurately predict the future per se (Beckert 2016, p. 218). It is especially important to understand whether and how forecasts achieve this goal, because by shaping expectations they may also drive behavioral changes, as evidenced by research on the effects of polls on voting behavior in multiparty systems (Van der Meer et al. 2016; Stolwijk et al. 2016; Dahlgaard et al. 2017)—though, more broadly, evidence on the behavioral effects of polls is mixed (Barnfield 2020; Roy et al. 2021).

Forecasts not only produce a statistical prediction of each candidate or party’s vote share, but also calculate their implied probabilities of winning the election—distilling a complex information environment into clear pictures of likely future outcomes. So-called “horse race” coverage simply portrays electoral contests in these terms (Toff 2019). To simplify things further still, forecasters also routinely provide a qualitative translation of this probability (e.g., somewhat/very/extremely likely). Notably, these approaches may be best suited to contexts where “winning” is relatively well defined, such as two-party majoritarian systems. In some contexts, the meaning of “victory” can be contingent on the electoral system and party size, such that it makes more sense to calculate a probability of passing a threshold for representation in parliament, entering into a governing coalition, or, as in our case, reaching the runoff round of a two-stage contest (Stiers et al. 2018; Plescia 2019).

Although vote shares and probabilities are just alternative presentations of the same underlying data, interpreting them as such when predicting the outcome may prove difficult. Achieving this feat with any precision would require knowing the variance of vote share estimates, along with a “relatively sophisticated background in statistics” (Westwood et al. 2020, p. 1532). People are also prone to cognitive biases when it comes to interpreting probabilities (Sunstein 2002; Szollosi et al. 2019). In addition, small changes in relative vote shares can result in much larger changes in implied probabilities of victory. All these factors are likely to confuse and complicate the translation between vote shares and probabilities.

People’s interpretations of verbal statements of probability are highly variable and context dependent, such that one person’s “quite likely” might be another person’s “somewhat likely” (Beyth-Marom 1982; Brun and Teigen 1988). And when repeatedly exposed to qualitative probability statements, people combine them differently from how they combine equivalent numerical probabilities (Mislavsky and Gaertig 2022). Such confusion can be offset by presenting numerical estimates alongside any verbal statement (Wintle et al. 2019). So it matters not only which type of information people get, but also whether and how it is combined with other types.

Voters can naturally express their expectations in the same terms as forecasts—as vote share predictions, numerical probabilities, or qualitative statements of likelihood (Manski 2004; Blais et al. 2008). When there is a match between forecasts and expectations, we might expect the former to be especially informative for the latter. However, voters may experience confusion if attempting to translate between them. Westwood et al. (2020) find that exposing voters to forecasts in the form of probabilities can lead them to considerably overstate a leading candidate’s chances in terms of vote share. Conversely, they find that vote share estimates lead voters to be less confident in stating which candidate will win.

In summary, the effect of forecasts on expectations is likely to depend on the form in which the forecast is presented, whether those forms are combined and presented in tandem, and the form the stated expectation takes.

We conducted a preregistered survey experiment via YouGov prior to the 2022 French presidential election to study these relationships. Unlike previous work (Madson and Hillygus 2020; Westwood et al. 2020; Leemann et al. 2021; Barnfield 2023b), we present voters with a real polling-based forecast (by *The Economist*) for a real and salient upcoming election, in a non-US context with more than two competing parties. This approach provides a balance of internal and external validity, making it more likely that the effects we observe generalize beyond the experimental context (Barnfield 2023a).

We find, indeed, that the format of both forecasts and survey items shapes the expectations voters express. Exposure to a forecast projecting vote shares consistently improves the accuracy (closer to the forecast itself and to the election result) and sometimes the precision (narrower distribution) of vote share expectations. In contrast, the same forecast in a probabilistic format sometimes makes these vote share expectations *less* accurate and *less* precise compared to when no information is provided. When combined, the effects of both formats tend to cancel out. On the other hand, both vote share information and probabilistic information taken from the same forecast, especially when combined, improve participants’ predictions of which candidates reach the second round. Qualitative statements tend to have fairly negligible effects. Our results demonstrate that while forecasts may be influential for expectations formation, there are key limits to how people process their results.

## Data and Methods

Our preregistered survey experiment took place immediately prior to the 2022 French presidential election (N = 2,934; April 1–8). The online survey was conducted by the polling firm YouGov, and uses matching and weighting to be nationally representative on key demographics (all analyses are unweighted).^1^ The Supplementary Material provides an overview of the demographics of the sample (Supplementary Material SM1), ethical approval information, preregistration, and data availability (Supplementary Material SM2). We estimate all effects through OLS models, reporting the results visually. Full tabulated summaries, including and excluding preregistered controls, are in Supplementary Material SM7 and SM8. Missing data is handled through listwise deletion.

### Experimental Design

**Figure 1.**
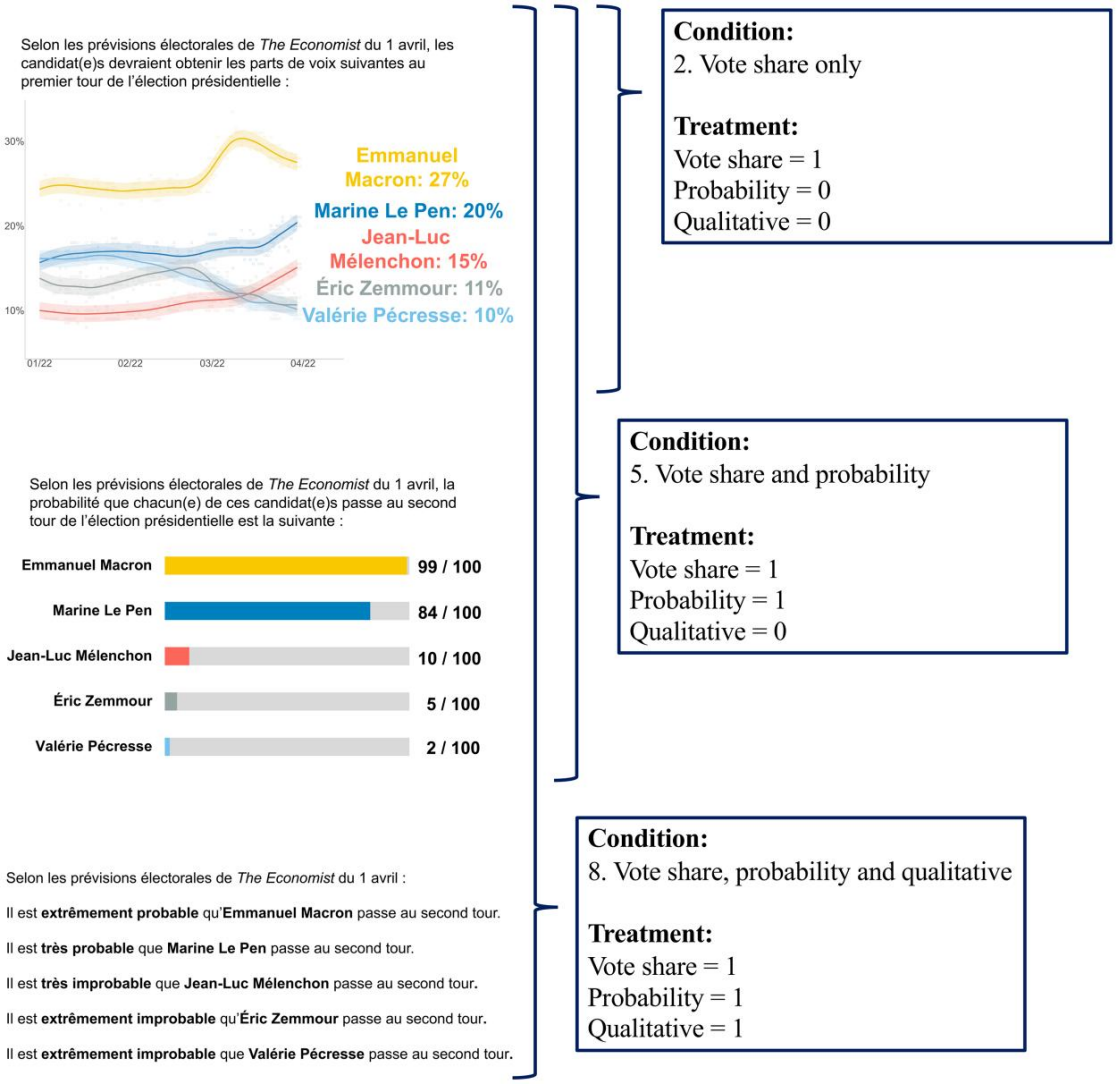
Forecast treatments and specification of independent variables. Top forecast format presents the candidates’ modeled average vote shares over time, up to April 1. Middle forecast format presents the candidates’ model-based probabilities of getting into the second round of the election. Bottom forecast format presents a qualitative interpretation of this probability. Respondents saw a random selection of random size, or none, of these formats. Boxes provide three examples of possible treatment assignments and how these correspond to our two different independent variable specifications.

We randomly exposed respondents to up to three separate formats of the same underlying forecast model. Some respondents saw no forecast, some saw only one format, some saw two, and some saw three. The forecast was taken, with permission, from *The Economist’*s French presidential election model, on April 1. Our three presentation formats mimic those presented in *The Economist’*s online coverage. Figure 1 shows how each format was presented. We provide full English translations of the text in the treatments in Supplementary Material SM5.

### Measures

#### Dependent variables

Our primary dependent variable is a measure of voters’ vote share expectations:

*In your opinion, what percentage of the vote will <candidate> receive in the first round?*

Respondents answer this question for three candidates. The first two are always Emmanuel Macron and Marine Le Pen, as they appeared most likely to make it to the second round. The third candidate rated was randomly assigned to be either Jean-Luc Mélenchon, Éric Zemmour, or Valérie Pécresse, to save survey time.

To measure accuracy, we use the difference between participants’ response to this question and (a) the true vote share underlying or reported in the forecast, and (b) the actual vote share achieved in the election.

To measure the precision of expectations, we take the difference between the lower and upper bound of the distribution of feasible vote shares elicited via two questions:

*Please indicate the [lowest/highest] percentage of the vote that you think <candidate> could receive in the first round.*

Respondents were prompted not to report higher/lower numbers than their predicted average vote share for these lower/upper bounds.

To assess predictions of which candidates would get into the second round, we asked respondents:

*Which two candidates will advance to the second round of the presidential election? Please choose two of the candidates from the list below, or specify an “other” candidate.*

Respondents who correctly predicted that Macron and Le Pen would advance are coded as 1, while those who failed to foresee this outcome are coded as 0.

#### Independent variables

We distinguish between two specifications of our experimental treatment variable, shown in figure 1 and table 1. Using a “condition” specification, we assess differences in outcomes between respondents across our total of eight possible conditions—ignoring different presentation orderings. Each condition represents a possible combination of forecast formats. These conditions are mutually exclusive. In our analyses, the baseline condition is 1. Pure control.

**Table 1.** Mutually exclusive experimental conditions/treatments. When a respondent sees more than one forecast, the order of presentation is randomized.

Number of forecasts	Condition	Treatment
0	1. Pure control	Vote share = 0, Probability = 0, Qualitative = 0
1	2. Vote share only	Vote share = 1, Probability = 0, Qualitative = 0
1	3. Probability only	Vote share = 0, Probability = 1, Qualitative = 0
1	4. Qualitative only	Vote share = 0, Probability = 0, Qualitative = 1
2	5. Vote share and probability	Vote share = 1, Probability = 1, Qualitative = 0
2	6. Vote share and qualitative	Vote share = 1, Probability = 0, Qualitative = 1
2	7. Probability and qualitative	Vote share = 0, Probability = 1, Qualitative = 1
3	8. Vote share, probability, and qualitative	Vote share = 1, Probability = 1, Qualitative = 1

In separate models, “treatment” estimates the effect of each forecast independently through three binary indicators of whether respondents were exposed to each forecast format, taking a value of 1 if the respondent received it and 0 if not. For example, the vote share forecast dummy takes the value 1 for a respondent who only received the vote share format, but also takes the value 1 for a respondent who received the vote share and probability formats.

To increase precision, our models all include controls for respondent gender, age, and education level (Bowers 2011). We also take measures of support for candidates and parties, political interest, and trust in experts as potential moderators of our effects (survey order shown in SM4). In SM10, we show that treatment effects vary minimally across these variables, though they themselves predict expectations, net of treatment.

## Results

**Figure 2.**
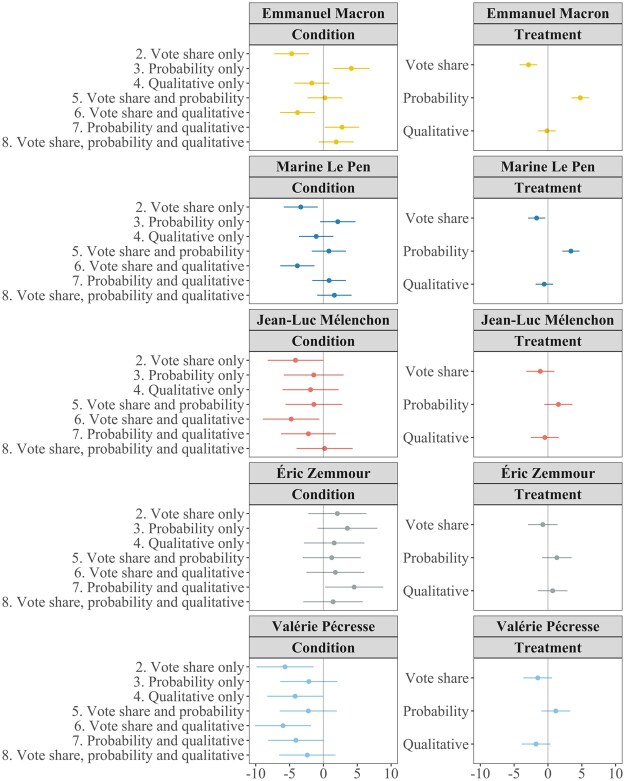
Average condition and treatment effects. Left column shows the average effect on vote share expectations of each condition (combination of forecast formats presented) compared to control (no forecast). Right column shows the independent average effect on expectations of each forecast format.

Figure 2 plots the marginal effect and 95 percent confidence interval of each condition relative to the pure control condition (left) and of each forecast treatment individually (right), on the raw reported vote share expectation (0–100) for each candidate.

When respondents see the vote share format, their vote share expectations tend to be lower; when they see the probabilistic format, they tend to be higher. These effects are most visible for Emmanuel Macron, for whom the decrease in expectations approaches 5 percentage points when respondents only see the vote share format (*p* < 0.001), or see it in tandem with a qualitative likelihood statement (*p =* 0.004). Conversely, expectations increase by a similar amount when respondents only see the probability format (*p =* 0.003), or see it in tandem with a qualitative likelihood statement (*p =* 0.035). These effects cancel out, such that any combination of vote share with the probabilistic format makes no discernible difference to expectations relative to the control group (*p =* 0.863), including when the qualitative format is also displayed (*p =* 0.155). For Marine Le Pen, the same tendencies emerge, except that the positive effects of the conditions including the probabilistic format are not statistically significant. However, for both leading candidates, the total effect of each of these two forecast formats is statistically significant. Averaging over different combinations, expectations are significantly lower for Emmanuel Macron (*p* < 0.001) and for Marine Le Pen (*p =* 0.011) when the vote share format is present, and significantly higher when the probabilistic forecast is present (both *p* < 0.001).

For the other three candidates, in most cases, these effects are indistinguishable from zero—with a few exceptions. For example, for Jean-Luc Mélenchon (*p =* 0.050) and Valérie Pécresse (*p =* 0.009), the vote share format significantly lowers expectations. In Supplementary Material SM10.1, we show that for Jean-Luc Mélenchon, this effect was largest among his supporters.

### Effects on Accuracy

**Figure 3.**
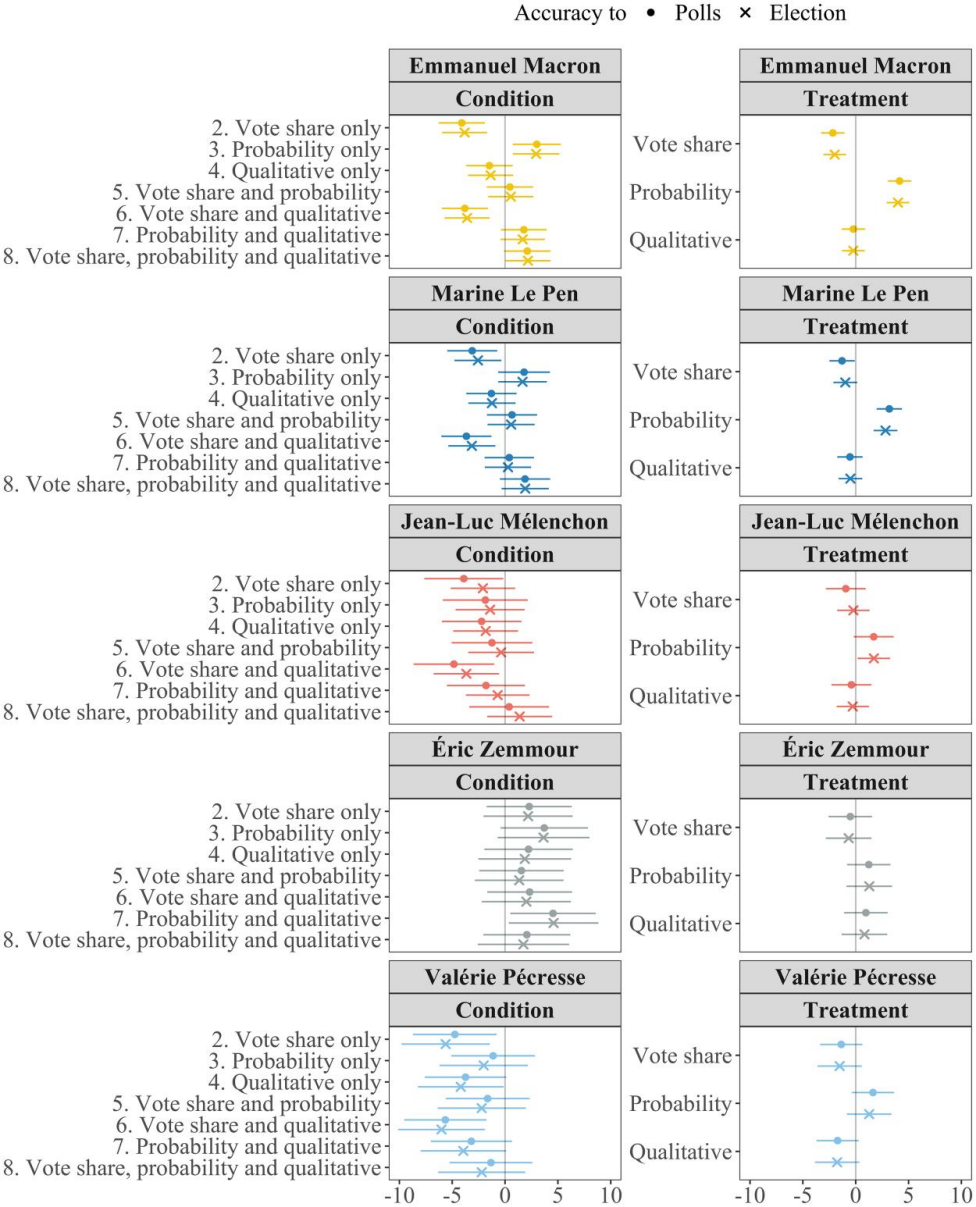
Condition and treatment effects on accuracy of vote share predictions. Left column shows the effect on the accuracy of vote share expectations of each condition (combination of forecast formats presented) compared to control (no forecast). Right column shows the independent effect on accuracy of expectations of each forecast format. Negative effects indicate that a condition/treatment increased accuracy.

As we show in Supplementary Material SM6, vote share expectations tend to be significant overestimates. So by lowering those expectations, vote share forecasts should bring them closer to reality, whereas probabilistic formats push them further away. Figure 3 assesses this possibility directly, by plotting the effects of the forecast formats on the absolute difference between voters’ expectations and, first, the vote share on which the forecast was based, and, second, the eventual election result. These accuracy effects confirm that vote share forecasts increase accuracy (that is, reducing absolute error) while probability forecasts decrease accuracy (increasing absolute error). Qualitative forecast formats appear to have little effect.

### Effects on Precision

**Figure 4.**
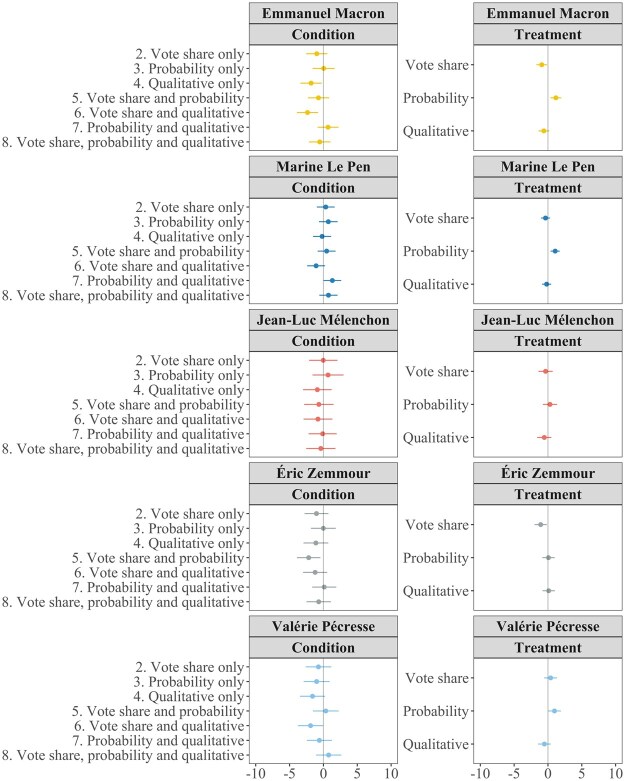
Condition and treatment effects on precision of vote share predictions. Left column shows the effect on the precision of vote share expectations of each condition (combination of forecast formats presented) compared to control (no forecast). Right column shows the independent effect on precision of expectations of each forecast format. Negative effects indicate that a condition/treatment increased precision.

Do forecasts affect the precision as well as the accuracy of expectations? For example, are those whose vote share expectations are pushed away from reality by a probabilistic format also less precise in their expectations? Figure 4 explores this possibility by plotting the effects of our forecast formats on the width of the range of vote shares implied by respondents’ reported upper and lower bounds.

For Emmanuel Macron, the qualitative format in isolation (*p =* 0.024), and combined with the vote share format (*p =* 0.004), narrows the range of plausible vote shares, increasing precision. For Éric Zemmour, the combination of the vote share and probability appears to significantly increase precision (*p =* 0.014). Such effects are not observed systematically across candidates, however.

The picture becomes clearer when looking at the total effects of each forecast format, in the right column of figure 4. Here, for Emmanuel Macron (*p =* 0.004), Marine Le Pen (*p =* 0.002), and Valérie Pécresse (*p =* 0.046), the total effect of the probability format is to widen the range of plausible vote shares—that is, to reduce precision. Meanwhile, for both Emmanuel Macron (*p =* 0.024) and Éric Zemmour (*p =* 0.021), the total effect of the vote share format is to increase precision by narrowing this range.

### Predicting the Second Round

**Figure 5.**
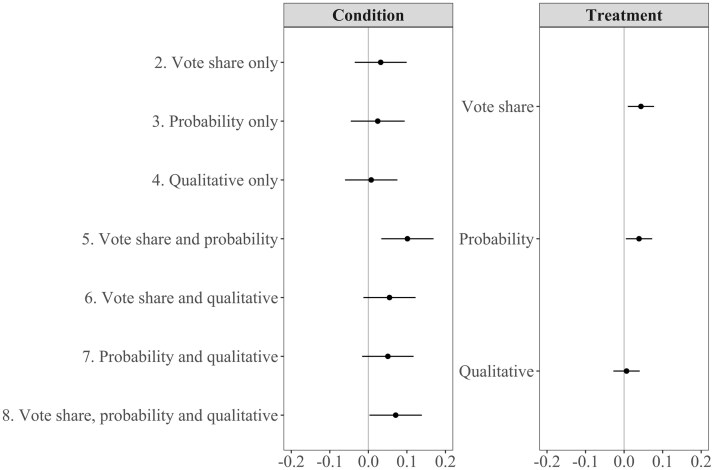
Condition and treatment effects on probability of predicting two correct candidates qualifying for second round. Left column shows the effect of each condition (combination of forecast formats presented) on the probability of predicting Emmanuel Macron and Marine Le Pen to quality for second round compared to control (no forecast). Right column shows the independent effect of each forecast format on correct predictions.

However, while probabilistic forecasts may not be as useful as vote share forecasts in helping people predict vote shares, that is not what they are designed to do. Rather, they are designed to distill that information into a prediction of who will win. Accordingly, figure 5 assesses the effect of our forecast formats on people’s ability to correctly predict which two candidates would get into the second round of the election (Emmanuel Macron and Marine Le Pen).

The only conditions that significantly improve the probability of correctly predicting the two candidates who will progress to the second round are those combining vote share and probability—with (*p =* 0.041) or without (*p =* 0.004) the addition of the qualitative likelihood statement. In Supplementary Material SM15, we also show that combining vote share and probability formats significantly reduced the time it took respondents to make this prediction (*p =* 0.008). Neither probabilistic nor vote share formats alone significantly improve second-round predictions. The total effects of displaying the vote share (*p =* 0.013) and probability (*p =* 0.027) formats—in the right panel of figure 5—are both significant and of equal size. Therefore, while probabilistic forecasts do appear to help people predict which candidates will win all else being equal, they do not outperform vote share forecasts in this regard, and may be insufficient in isolation.

## Discussion

In a highly salient real election context, a real forecast had substantial effects on voters’ electoral expectations. In line with recent work (Stoetzer et al. 2024), we show that the significant effects of polls and polling-based forecasts on expectations observed in abstract or hypothetical experimental studies generalizes to real-world elections (e.g., Westwood et al. 2020; Leemann et al. 2021; Barnfield 2023b).

When presented as projected vote shares, our forecast pushed voters toward more accurate, sometimes more precise vote share expectations. A meaningful causal effect likely underpins the relationship reported in observational studies between polls and accurate expectations (Lavrakas et al. 1991; Irwin and Van Holsteyn 2002; Zerback et al. 2015; Bowler et al. 2022).

In contrast, where it had an effect, a probabilistic presentation of this forecast decreased accuracy, consistent with abstract experimental work in the United States (Westwood et al. 2020). Clearly, vote share forecasts are well suited to the task of predicting vote shares, as the information and the stated expectation match exactly. However, not only is our probabilistic format understandably outperformed by the vote share format, but the probabilistic format performs as badly or worse than *no* forecast information. Whereas past work has suggested that probabilistic forecasts increase certainty about election results, their effect on precision in our study suggests that probabilities make people less certain about likely vote shares (Westwood et al. 2020). Therefore, while our findings echo the commonly expressed concern that probabilistic forecasts confuse people’s expectations, they also demonstrate new dimensions of this effect in contexts beyond where it is usually studied (Pentzold and Fechner 2020; Westwood et al. 2020; Victor 2021).

We bring further nuance to this conclusion by showing that probabilistic forecasts help voters in accurately predicting the winner—arguably, what they are designed to do. Previous work has demonstrated that probabilistic forecasts raise expectations of the leading party’s chances in the abstract, but our use of a real election verifies that this ultimately increases correct predictions of the eventual outcome (Westwood et al. 2020). However, our vote share format appears to be equally helpful for this purpose, with the combination of the two proving particularly informative.

These nuanced insights into the different effects of forecasts call for, and could inform, normative debate about the intended role of forecasts in election coverage. Scholars should discuss the importance of accurate expectations and whether it is more desirable for voters to feel more certainty about precise outcomes, or rather entertain a wider range of possibilities. The value of forecasting hinges on the answers to such questions.

Future work should also seek to address some limitations of the present study. Namely, although we have attempted to rule out a range of moderators of the effects we observe, others—such as levels of political sophistication, existing electoral knowledge, or numerical literacy—could have an influence on the reception of forecast information outside of our particular experimental context (Zerback et al. 2022; Zaller 1992). Additionally, the effects we observe may vary in nonelectoral forms of forecasting. This possibility calls for a broader program of research into how forecasts are interpreted across a range of social, political, and economic domains.

## Supplementary Material

nfaf003_Supplementary_Data

## Data Availability

Replication data and documentation are available at https://osf.io/yaqh7/?view_only=5026700a78e741ac894f772420e9e896.

## Footnotes

1 As this survey uses a nonprobability sample with participants selected from the YouGov panel rather than being directly invited, a conventional response rate cannot be provided.
