# Supplementary material for: The Effects of Forecasts on the Accuracy and Precision of Expectations
Source: Public Opin Q. 2025 May 9;89(1):185–200. doi: 10.1093/poq/nfaf003 (PMC12166975; doi:10.1093/poq/nfaf003)
Supplement: nfaf003_Supplementary_Data [file nfaf003_supplementary_data.pdf]

Supplementary Material: The Effects of Forecasts on the Accuracy and Precision of Expectations

Matthew Barnfield, School of Politics and International Relations, Queen Mary University of London, m.g.barnfield@gmail.com; **Joseph Phillips**, School of Psychology, University of Kent, j.phillips-823@kent.ac.uk; **Florian Stoeckel**, Department of Politics, University of Exeter, f.stoeckel@exeter.ac.uk; **Benjamin Lyons**, Department of Communication, University of Utah, Ben.lyons@utah.edu; **Paula Szewach**, Barcelona Supercomputing Centre, paula.szewach@bsc.es; **Jack Thompson**, Leeds University Business School, University of Leeds, j.thompson@leeds.ac.uk; **Vittorio Mérola**, School of Government and International Affairs, Durham University, vittorio.merola@durham.ac.uk; **Sabrina Stöckli**, Chair of Marketing, University of Zurich, sabrina.stoeckli@business.uzh.ch; **Jason Reifler**, Department of Politics, University of Exeter, j.reifler@exeter.ac.uk.

Contents

SM1 Sample Composition 1

SM2 Ethics, Pre-registration, Data Availability 2

SM3 Pre-Registered Research Questions 2

SM4 Order of Questionnaire 4

SM5 English Translations of Forecast Treatments 5

- **SM5.1** Vote Share 5
- **SM5.2** Probabilistic 5
- **SM5.3** Qualitative 5

SM6 Distribution of expectations by condition 6

SM7 Main model tables 8

SM8 Main models without controls 19

SM9 Randomisation Check 31

SM10 Effect Heterogeneity 33

- **SM10.1** Candidate Preferences 33
- **SM10.2** Political Interest 41
- **SM10.3** Anti-expert Sentiment/Self-efficacy 43

SM11 Treatment interactions 44

SM12 Effects on Vote Choice 48

SM13 Top-Two Advantage 53

SM14 Alternative Second Round Prediction Specification 55

SM15 Response Time Model for Second Round Prediction 57

SM16 Variation in Effects Over Time 58

SM17 Manski Questions 60

SM18 Interaction Effect Benjamini Hochberg-Adjusted P-Values 61

Supplementary Material

SM1 Sample Composition

Table SM1: Sample composition.

	N	Percentage
**Gender**		
Male	1335	45.50
Female	1599	54.50
**Age**		
Under 25	237	8.08
25-44	808	27.54
45-54	535	18.23
55+	1354	46.15
**Education**		
No university	1781	60.70
University	1153	39.30
**Region**		
Grand Est	258	8.79
Nouvelle Aquitaine	253	8.62
Auvergne, Rhône-Alpes	360	12.27
Normandie	176	6.00
Bourgogne, Franche-Comté	101	3.44
Bretagne	131	4.46
Centre- Val de Loire	118	4.02
Ile-de-France	556	18.95
Occitanie	288	9.82
Haut de France	313	10.67
Pays de la Loire	153	5.21
Provence-Alpes Côte d'Azur	227	7.74

SM2 Ethics, Pre-registration, Data Availability

We obtained ethical approval for this study from a major UK University (blinded for review). The research complies with General Data Protection Regulation requirements. The data were collected, and made available on OSF without identifying information, and with informed consent from the respondents. We also pre-registered research questions, primary, and secondary analyses before receiving any of our data from YouGov on 29th April, 2022 at OSF (anonymized pre-registration link: `https://osf.io/4xagr/?view_only=ad61abab41b04a87aeb7a8585c792484`). We provide our data and code on OSF (anonymized data and code link: `https://osf.io/yaqh7/?view_only=0c48a4b83c7049238dd406e59839f224`).

SM3 Pre-Registered Research Questions

- How do different types and different combinations of forecasts affect vote share expectations on average?
  - Addressed in Average Effects section of main text, and visually in Figure 2.
- How do different types and different combinations of forecasts affect accuracy of vote share expectations, in terms of proximity to each candidate's predicted performance?
  - Addressed in Effects on Accuracy section of main text, and visually in Figure 3.
- How do different types and different combinations of forecasts affect accuracy of vote share expectations, in terms of proximity to the eventual actual performance of each candidate in the election?
  - Addressed in Effects on Accuracy section of main text, and visually in Figure 3.
- How do different types and different combinations of forecasts affect the precision of vote share expectations, in terms of the spread of the distribution of perceived probability?

- Addressed in Effects on Precision section of main text, and visually in Figure 4.

- How do different types and different combinations of forecasts affect whether voters predict the eventual election winner(s)?
  - Addressed in Predicting the Second Round section of main text, and visually in Figure 5. Also addressed in section SM14 of Supplementary Material.

- How do different types and different combinations of forecasts affect voting intentions?
  - Addressed in section SM12 of Supplementary Material, and visually in Figure SM4.

- How do electoral expectations, and the effects of different types and combinations of forecasts on these expectations, vary by measures of political support/preference (ideological distance, feelings towards parties, party identification)?
  - Addressed in section SM10.1, Table SM25, Table SM26, and Table SM27.

- How do electoral expectations, and the effects of different types and combinations of forecasts on these expectations, vary by levels of political interest?
  - Addressed in section SM10.2 and Table SM28.

- How do electoral expectations, and the effects of different types and combinations of forecasts on these expectations, vary by levels of trust in expert opinion?
  - Addressed in section SM10.3 and Table SM29.

- How do electoral expectations, and the effects of different types and combinations of forecasts on these expectations, vary over time?
  - Addressed in section SM16.

SM4 Order of Questionnaire

Figure SM1: Flow of YouGov survey.

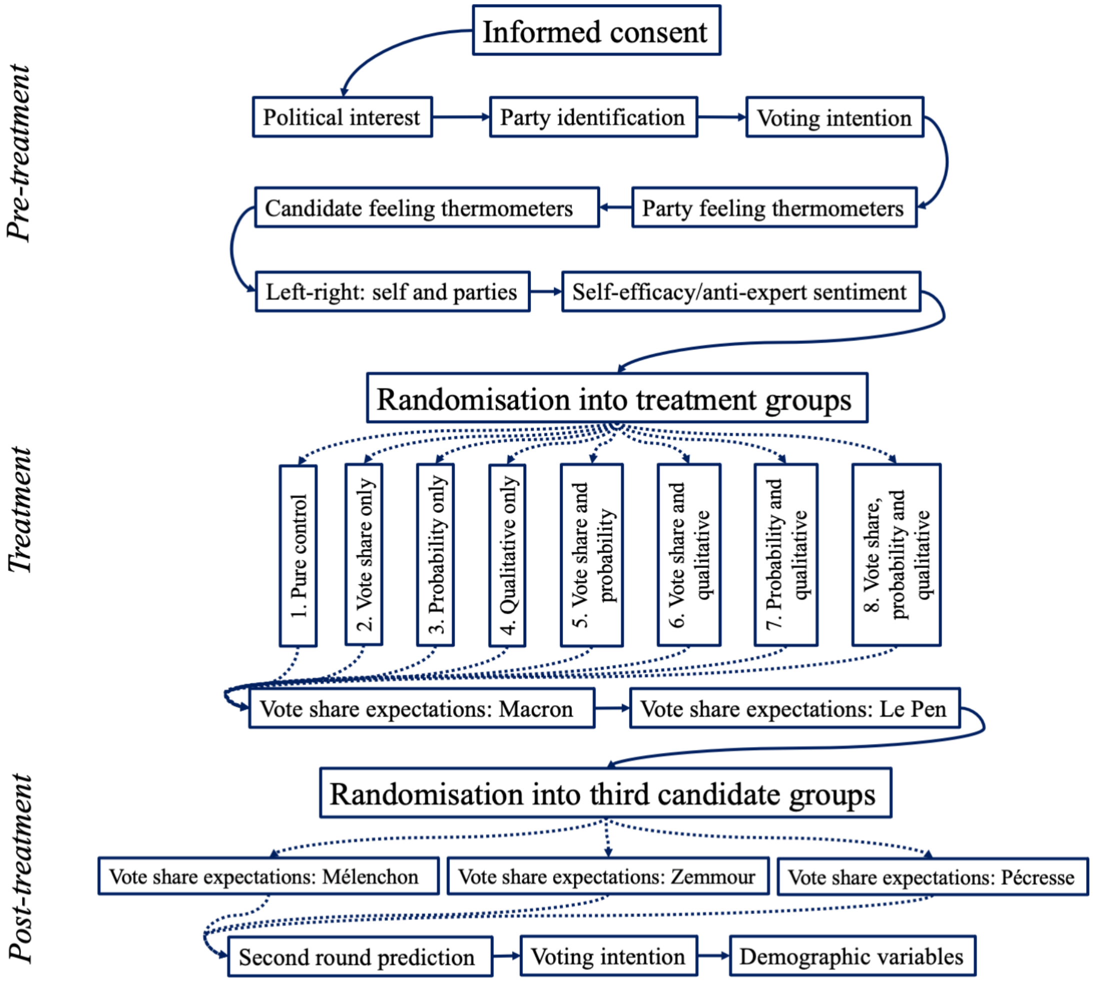

Note. Diagram showing the order of items presented in our online survey, only including those survey items used in our analyses. Our survey was part of a multi-study project and therefore included other items not relevant to the present study that are not displayed here.

SM5 English Translations of Forecast Treatments

SM5.1 Vote Share

According to *The Economist*'s electoral forecast on 1st April, the candidates should receive the following vote shares in the first round of the presidential election.

SM5.2 Probabilistic

According to *The Economist*'s electoral forecast on 1st April, the probability that each of the candidates advances to the second round of the presidential election is as follows.

SM5.3 Qualitative

According to *The Economist*'s electoral forecast on 1st April:

It is extremely likely that Emmanuel Macron advances to the second round.

It is very likely that Marine Le Pen advances to the second round.

It is very unlikely that Jean-Luc Mélenchon advances to the second round.

It is extremely unlikely that Éric Zemmour advances to the second round.

It is extremely unlikely that Valérie Pécresse advances to the second round.

SM6 Distribution of expectations by condition

Figure SM2 plots the average predicted vote share for each candidate, in each condition. For all candidates, average vote share expectations are considerably higher both than polls at the time suggested they should be and than the vote shares the candidates eventually received in the election. For example, in the pure control condition, on average respondents predicted that Macron would get approximately 40% of the vote—well over his eventual total of around 28%. This discrepancy was largest for Valérie Pécresse, unanimously predicted to secure around 20% of the vote, when in fact she only got 5% in the election. Beyond this general over-estimation, broadly speaking, conditions featuring a probabilistic forecast seem to elicit higher expected vote shares, while conditions featuring vote share forecasts elicit lower expected vote shares.

Figure SM2: Summary of expectations by condition.

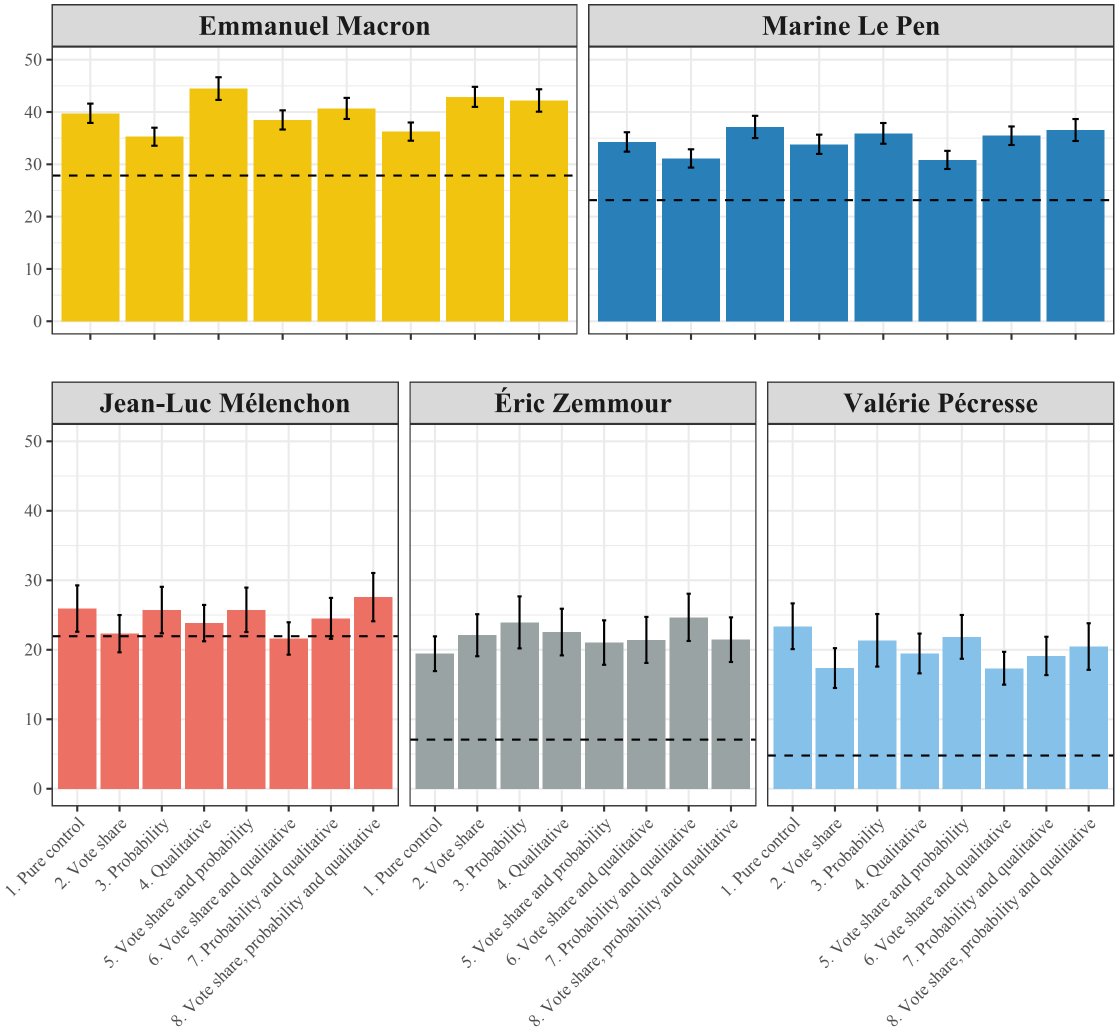

Note. Bars plot average predicted vote share in each condition, error bars display 95% confidence intervals. Dashed horizontal lines display each candidate's actual vote share achieved in the election.

SM7 Main model tables

Tables SM3-SM11 provide full summaries of models reported in the main text: effects of condition and treatment independent variable specifications on average vote share expectations, on accuracy of vote share expectations, on precision of vote share expectations, and on correctly predicting which candidates would reach the second round.

Table SM2: Effects of condition on vote share expectations

	Dependent variable:				
	Emmanuel Macron	Marine Le Pen	Jean-Luc Mélenchon	Éric Zemmour	Valérie Pécresse
	(1)	(2)	(3)	(4)	(5)
Constant	45.243 (1.556)	41.724 (1.523)	35.993 (2.511)	26.876 (2.601)	31.839 (2.534)
	p = 0.000	p = 0.000	p = 0.000	p = 0.000	p = 0.000
**Condition**					
Vote share only	−4.693 (1.310)	−3.352 (1.283)	−4.124 (2.092)	2.056 (2.187)	−5.676 (2.148)
	p = 0.0004	p = 0.010	p = 0.050	p = 0.348	p = 0.009
Probability only	4.129 (1.359)	2.120 (1.330)	−1.434 (2.248)	3.543 (2.252)	−2.155 (2.148)
	p = 0.003	p = 0.112	p = 0.524	p = 0.116	p = 0.317
Qualitative only	−1.716 (1.321)	−1.068 (1.293)	−1.901 (2.108)	1.587 (2.275)	−4.183 (2.093)
	p = 0.195	p = 0.410	p = 0.368	p = 0.486	p = 0.046
Vote share and probability	0.229 (1.317)	0.805 (1.289)	−1.412 (2.138)	1.232 (2.175)	−2.236 (2.149)
	p = 0.863	p = 0.533	p = 0.510	p = 0.572	p = 0.299
Vote share and qualitative	−3.832 (1.315)	−3.855 (1.287)	−4.769 (2.129)	1.763 (2.182)	−5.971 (2.111)
	p = 0.004	p = 0.003	p = 0.026	p = 0.420	p = 0.005
Probability and qualitative	2.747 (1.296)	0.834 (1.269)	−2.221 (2.065)	4.546 (2.197)	−4.065 (2.085)
	p = 0.035	p = 0.511	p = 0.283	p = 0.039	p = 0.052
Vote share, probability and qualitative	1.882 (1.320)	1.618 (1.294)	0.174 (2.112)	1.426 (2.239)	−2.382 (2.122)
	p = 0.155	p = 0.212	p = 0.935	p = 0.525	p = 0.262
**Controls**					
Gender	6.423 (0.670)	5.052 (0.656)	3.633 (1.069)	2.716 (1.133)	0.890 (1.091)
	p = 0.000	p = 0.000	p = 0.001	p = 0.017	p = 0.416
University	−4.496 (0.699)	−7.962 (0.685)	−4.809 (1.100)	−6.253 (1.185)	−3.476 (1.151)
	p = 0.000	p = 0.000	p = 0.00002	p = 0.00000	p = 0.003
Age 25-44	−2.655 (1.329)	−2.575 (1.301)	−6.726 (2.089)	0.007 (2.208)	−3.666 (2.240)
	p = 0.046	p = 0.048	p = 0.002	p = 0.998	p = 0.103
Age 45-54	−5.302 (1.406)	−4.792 (1.377)	−8.798 (2.264)	−5.984 (2.299)	−5.947 (2.352)
	p = 0.0002	p = 0.001	p = 0.0002	p = 0.010	p = 0.012
Age 55+	−11.104 (1.280)	−10.876 (1.253)	−13.293 (2.040)	−10.298 (2.106)	−11.634 (2.148)
	p = 0.000	p = 0.000	p = 0.000	p = 0.00001	p = 0.00000
Observations	2,934	2,933	1,000	989	942
R^2	0.109	0.109	0.085	0.088	0.072
Adjusted R^2	0.105	0.105	0.074	0.077	0.060

Table SM3: Effects of treatment on vote share expectations

	Dependent variable:				
	Emmanuel Macron	Marine Le Pen	Jean-Luc Mélenchon	Éric Zemmour	Valérie Pécresse
	(1)	(2)	(3)	(4)	(5)
Constant	44.142 (1.409)	40.743 (1.380)	34.017 (2.272)	28.344 (2.301)	29.759 (2.343)
	p = 0.000	p = 0.000	p = 0.000	p = 0.000	p = 0.000
**Treatment**					
Vote share	−2.886 (0.664)	−1.675 (0.650)	−1.128 (1.053)	−0.761 (1.118)	−1.509 (1.082)
	p = 0.00002	p = 0.011	p = 0.285	p = 0.497	p = 0.164
Probability	4.774 (0.664)	3.399 (0.651)	1.539 (1.057)	1.313 (1.118)	1.163 (1.084)
	p = 0.000	p = 0.00000	p = 0.146	p = 0.241	p = 0.284
Qualitative	−0.155 (0.664)	−0.548 (0.651)	−0.463 (1.053)	0.692 (1.120)	−1.764 (1.084)
	p = 0.817	p = 0.400	p = 0.661	p = 0.537	p = 0.105
**Controls**					
Gender	6.452 (0.670)	5.052 (0.656)	3.586 (1.069)	2.676 (1.131)	0.861 (1.093)
	p = 0.000	p = 0.000	p = 0.001	p = 0.019	p = 0.431
University	−4.505 (0.699)	−7.989 (0.684)	−4.766 (1.099)	−6.274 (1.182)	−3.688 (1.146)
	p = 0.000	p = 0.000	p = 0.00002	p = 0.00000	p = 0.002
Age 25-44	−2.604 (1.328)	−2.517 (1.301)	−6.572 (2.084)	−0.006 (2.201)	−3.783 (2.238)
	p = 0.050	p = 0.054	p = 0.002	p = 0.998	p = 0.092
Age 45-54	−5.278 (1.406)	−4.794 (1.377)	−8.840 (2.264)	−5.941 (2.290)	−6.067 (2.348)
	p = 0.0002	p = 0.001	p = 0.0002	p = 0.010	p = 0.010
Age 55+	−11.024 (1.280)	−10.831 (1.253)	−13.270 (2.039)	−10.387 (2.096)	−11.668 (2.143)
	p = 0.000	p = 0.000	p = 0.000	p = 0.00000	p = 0.00000
Observations	2,934	2,933	1,000	989	942
R^2	0.107	0.107	0.079	0.085	0.065
Adjusted R^2	0.105	0.105	0.071	0.078	0.057

Table SM4: Effects of condition on accuracy of vote share expectations (relative to current polling)

	Dependent variable:				
	Emmanuel Macron	Marine Le Pen	Jean-Luc Mélenchon	Éric Zemmour	Valérie Pécresse
	(1)	(2)	(3)	(4)	(5)
Constant	21.696 (1.322)	23.709 (1.431)	21.775 (2.287)	17.762 (2.438)	22.812 (2.381)
	p = 0.000	p = 0.000	p = 0.000	p = 0.000	p = 0.000
**Condition**					
Vote share only	−4.091 (1.113)	−3.109 (1.205)	−3.903 (1.906)	2.283 (2.050)	−4.750 (2.018)
	p = 0.0003	p = 0.010	p = 0.041	p = 0.266	p = 0.019
Probability only	3.001 (1.154)	1.799 (1.249)	−1.865 (2.048)	3.711 (2.111)	−1.129 (2.018)
	p = 0.010	p = 0.151	p = 0.363	p = 0.080	p = 0.577
Qualitative only	−1.480 (1.122)	−1.292 (1.215)	−2.216 (1.920)	2.218 (2.132)	−3.741 (1.966)
	p = 0.188	p = 0.288	p = 0.249	p = 0.299	p = 0.058
Vote share and probability	0.455 (1.118)	0.656 (1.211)	−1.237 (1.948)	1.548 (2.039)	−1.652 (2.019)
	p = 0.684	p = 0.589	p = 0.526	p = 0.448	p = 0.414
Vote share and qualitative	−3.807 (1.117)	−3.656 (1.209)	−4.841 (1.940)	2.330 (2.046)	−5.650 (1.983)
	p = 0.001	p = 0.003	p = 0.013	p = 0.256	p = 0.005
Probability and qualitative	1.777 (1.101)	0.399 (1.192)	−1.807 (1.881)	4.539 (2.060)	−3.193 (1.959)
	p = 0.107	p = 0.738	p = 0.338	p = 0.028	p = 0.104
Vote share, probability and qualitative	2.101 (1.121)	1.886 (1.215)	0.386 (1.924)	2.061 (2.098)	−1.339 (1.993)
	p = 0.062	p = 0.121	p = 0.841	p = 0.327	p = 0.502
**Controls**					
Gender	5.285 (0.569)	5.059 (0.616)	3.793 (0.974)	2.571 (1.062)	1.177 (1.025)
	p = 0.000	p = 0.000	p = 0.0002	p = 0.016	p = 0.252
University	−4.898 (0.594)	−7.878 (0.643)	−4.213 (1.002)	−6.355 (1.111)	−3.891 (1.082)
	p = 0.000	p = 0.000	p = 0.00003	p = 0.000	p = 0.0004
Age 25-44	−1.604 (1.128)	−3.248 (1.222)	−5.337 (1.903)	−0.853 (2.070)	−3.570 (2.105)
	p = 0.156	p = 0.008	p = 0.006	p = 0.681	p = 0.091
Age 45-54	−4.915 (1.194)	−5.555 (1.293)	−7.209 (2.062)	−5.903 (2.155)	−6.247 (2.210)
	p = 0.00004	p = 0.00002	p = 0.0005	p = 0.007	p = 0.005
Age 55+	−10.432 (1.087)	−11.883 (1.177)	−12.668 (1.858)	−10.947 (1.974)	−12.002 (2.018)
	p = 0.000	p = 0.000	p = 0.000	p = 0.00000	p = 0.000
Observations	2,934	2,933	1,000	989	942
R^2	0.128	0.125	0.102	0.099	0.086
Adjusted R^2	0.124	0.121	0.091	0.088	0.075

Table SM5: Effects of treatment on accuracy of vote share expectations (relative to current polling)

	Dependent variable:				
	Emmanuel Macron	Marine Le Pen	Jean-Luc Mélenchon	Éric Zemmour	Valérie Pécresse
	(1)	(2)	(3)	(4)	(5)
Constant	20.490 (1.197)	22.575 (1.297)	19.642 (2.071)	19.332 (2.157)	20.964 (2.201)
	p = 0.000	p = 0.000	p = 0.000	p = 0.000	p = 0.000
**Treatment**					
Vote share	−2.161 (0.564)	−1.289 (0.611)	−0.934 (0.960)	−0.512 (1.048)	−1.366 (1.016)
	p = 0.0002	p = 0.035	p = 0.331	p = 0.626	p = 0.179
Probability	4.154 (0.565)	3.181 (0.611)	1.706 (0.963)	1.239 (1.048)	1.635 (1.018)
	p = 0.000	p = 0.00000	p = 0.077	p = 0.238	p = 0.109
Qualitative	−0.219 (0.565)	−0.541 (0.611)	−0.407 (0.960)	0.965 (1.050)	−1.713 (1.018)
	p = 0.698	p = 0.376	p = 0.672	p = 0.359	p = 0.093
**Controls**					
Gender	5.302 (0.569)	5.062 (0.616)	3.747 (0.974)	2.529 (1.061)	1.156 (1.026)
	p = 0.000	p = 0.000	p = 0.0002	p = 0.018	p = 0.261
University	−4.913 (0.594)	−7.902 (0.643)	−4.147 (1.002)	−6.389 (1.108)	−4.060 (1.077)
	p = 0.000	p = 0.000	p = 0.00004	p = 0.000	p = 0.0002
Age 25-44	−1.543 (1.129)	−3.179 (1.222)	−5.209 (1.900)	−0.867 (2.063)	−3.620 (2.102)
	p = 0.172	p = 0.010	p = 0.007	p = 0.675	p = 0.086
Age 45-54	−4.900 (1.195)	−5.551 (1.294)	−7.281 (2.064)	−5.882 (2.147)	−6.293 (2.206)
	p = 0.00005	p = 0.00002	p = 0.0005	p = 0.007	p = 0.005
Age 55+	−10.360 (1.088)	−11.824 (1.177)	−12.661 (1.859)	−11.050 (1.965)	−11.972 (2.013)
	p = 0.000	p = 0.000	p = 0.000	p = 0.00000	p = 0.000
Observations	2,934	2,933	1,000	989	942
R^2	0.125	0.122	0.095	0.096	0.080
Adjusted R^2	0.123	0.120	0.087	0.088	0.072

Table SM6: Effects of condition on accuracy of vote share expectations (relative to election result)

	Dependent variable:				
	Emmanuel Macron	Marine Le Pen	Jean-Luc Mélenchon	Éric Zemmour	Valérie Pécresse
	(1)	(2)	(3)	(4)	(5)
Constant	21.157 (1.287)	21.994 (1.344)	18.433 (1.861)	20.337 (2.558)	26.984 (2.506)
	p = 0.000	p = 0.000	p = 0.000	p = 0.000	p = 0.000
**Condition**					
Vote share only	−3.824 (1.084)	−2.568 (1.132)	−2.094 (1.551)	2.176 (2.151)	−5.623 (2.124)
	p = 0.0005	p = 0.024	p = 0.178	p = 0.312	p = 0.009
Probability only	2.938 (1.124)	1.655 (1.174)	−1.413 (1.667)	3.640 (2.215)	−2.018 (2.125)
	p = 0.009	p = 0.159	p = 0.397	p = 0.101	p = 0.343
Qualitative only	−1.368 (1.093)	−1.242 (1.141)	−1.834 (1.563)	1.869 (2.237)	−4.191 (2.070)
	p = 0.211	p = 0.277	p = 0.241	p = 0.404	p = 0.044
Vote share and probability	0.547 (1.089)	0.574 (1.137)	−0.369 (1.585)	1.337 (2.139)	−2.212 (2.125)
	p = 0.616	p = 0.614	p = 0.816	p = 0.532	p = 0.299
Vote share and qualitative	−3.594 (1.088)	−3.142 (1.135)	−3.663 (1.579)	2.014 (2.146)	−6.014 (2.088)
	p = 0.001	p = 0.006	p = 0.021	p = 0.349	p = 0.005
Probability and qualitative	1.672 (1.072)	0.278 (1.119)	−0.699 (1.531)	4.592 (2.161)	−3.948 (2.062)
	p = 0.120	p = 0.805	p = 0.648	p = 0.034	p = 0.056
Vote share, probability and qualitative	2.163 (1.092)	1.904 (1.141)	1.381 (1.566)	1.736 (2.202)	−2.207 (2.099)
	p = 0.048	p = 0.096	p = 0.379	p = 0.431	p = 0.294
**Controls**					
Gender	4.964 (0.554)	4.332 (0.578)	1.618 (0.792)	2.793 (1.115)	0.929 (1.080)
	p = 0.000	p = 0.000	p = 0.042	p = 0.013	p = 0.390
University	−4.732 (0.579)	−7.077 (0.604)	−2.038 (0.816)	−6.376 (1.165)	−3.636 (1.139)
	p = 0.000	p = 0.000	p = 0.013	p = 0.00000	p = 0.002
Age 25-44	−1.440 (1.099)	−2.980 (1.147)	−3.131 (1.549)	−0.352 (2.172)	−3.561 (2.216)
	p = 0.191	p = 0.010	p = 0.044	p = 0.872	p = 0.109
Age 45-54	−4.722 (1.163)	−5.392 (1.214)	−4.516 (1.678)	−6.058 (2.261)	−5.921 (2.326)
	p = 0.0001	p = 0.00001	p = 0.008	p = 0.008	p = 0.012
Age 55+	−10.065 (1.059)	−11.230 (1.106)	−8.528 (1.512)	−10.727 (2.071)	−11.673 (2.125)
	p = 0.000	p = 0.000	p = 0.00000	p = 0.00000	p = 0.00000
Observations	2,934	2,933	1,000	989	942
R^2	0.125	0.118	0.073	0.094	0.075
Adjusted R^2	0.121	0.114	0.061	0.083	0.063

Table SM7: Effects of treatment on accuracy of vote share expectations (relative to election result)

	Dependent variable:				
	Emmanuel Macron	Marine Le Pen	Jean-Luc Mélenchon	Éric Zemmour	Valérie Pécresse
	(1)	(2)	(3)	(4)	(5)
Constant	20.015 (1.166)	20.958 (1.218)	16.763 (1.686)	21.853 (2.263)	24.890 (2.318)
	p = 0.000	p = 0.000	p = 0.000	p = 0.000	p = 0.000
**Treatment**					
Vote share	−1.987 (0.550)	−0.985 (0.574)	−0.216 (0.781)	−0.666 (1.099)	−1.517 (1.070)
	p = 0.0004	p = 0.087	p = 0.783	p = 0.545	p = 0.157
Probability	4.002 (0.550)	2.821 (0.574)	1.718 (0.784)	1.289 (1.099)	1.277 (1.073)
	p = 0.000	p = 0.00000	p = 0.029	p = 0.241	p = 0.235
Qualitative	−0.222 (0.550)	−0.499 (0.574)	−0.277 (0.781)	0.832 (1.102)	−1.757 (1.072)
	p = 0.687	p = 0.385	p = 0.724	p = 0.451	p = 0.102
**Controls**					
Gender	4.980 (0.554)	4.336 (0.579)	1.571 (0.793)	2.752 (1.113)	0.901 (1.081)
	p = 0.000	p = 0.000	p = 0.048	p = 0.014	p = 0.405
University	−4.749 (0.578)	−7.097 (0.604)	−1.976 (0.816)	−6.401 (1.163)	−3.844 (1.134)
	p = 0.000	p = 0.000	p = 0.016	p = 0.00000	p = 0.001
Age 25-44	−1.377 (1.099)	−2.910 (1.148)	−3.052 (1.547)	−0.364 (2.165)	−3.665 (2.214)
	p = 0.211	p = 0.012	p = 0.049	p = 0.867	p = 0.099
Age 45-54	−4.706 (1.164)	−5.382 (1.215)	−4.601 (1.680)	−6.022 (2.252)	−6.028 (2.323)
	p = 0.0001	p = 0.00001	p = 0.007	p = 0.008	p = 0.010
Age 55+	−9.994 (1.059)	−11.169 (1.106)	−8.546 (1.513)	−10.820 (2.062)	−11.691 (2.120)
	p = 0.000	p = 0.000	p = 0.00000	p = 0.00000	p = 0.00000
Observations	2,934	2,933	1,000	989	942
R^2	0.122	0.115	0.065	0.091	0.068
Adjusted R^2	0.120	0.113	0.057	0.083	0.060

Table SM8: Effects of condition on precision of vote share expectations

	Dependent variable:				
	Emmanuel Macron	Marine Le Pen	Jean-Luc Mélenchon	Éric Zemmour	Valérie Pécresse
	(1)	(2)	(3)	(4)	(5)
Constant	19.063 (0.973)	15.720 (0.831)	14.300 (1.340)	12.912 (1.113)	13.524 (1.157)
	p = 0.000	p = 0.000	p = 0.000	p = 0.000	p = 0.000
**Condition**					
Vote share only	−0.983 (0.794)	0.344 (0.672)	−0.025 (1.068)	−1.027 (0.899)	−0.735 (0.967)
	p = 0.216	p = 0.610	p = 0.982	p = 0.254	p = 0.448
Probability only	0.041 (0.828)	0.721 (0.704)	0.679 (1.167)	−0.012 (0.934)	−1.004 (0.977)
	p = 0.961	p = 0.306	p = 0.561	p = 0.990	p = 0.305
Qualitative only	−1.823 (0.805)	−0.197 (0.680)	−0.898 (1.075)	−1.115 (0.933)	−1.615 (0.944)
	p = 0.024	p = 0.773	p = 0.405	p = 0.233	p = 0.088
Vote share and probability	−0.732 (0.800)	0.475 (0.674)	−0.676 (1.105)	−2.176 (0.882)	0.347 (0.966)
	p = 0.361	p = 0.481	p = 0.541	p = 0.014	p = 0.720
Vote share and qualitative	−2.349 (0.794)	−1.080 (0.672)	−0.808 (1.084)	−1.223 (0.905)	−1.897 (0.945)
	p = 0.004	p = 0.109	p = 0.457	p = 0.178	p = 0.045
Probability and qualitative	0.678 (0.788)	1.322 (0.665)	−0.114 (1.056)	0.118 (0.908)	−0.598 (0.940)
	p = 0.390	p = 0.047	p = 0.914	p = 0.897	p = 0.525
Vote share, probability and qualitative	−0.549 (0.811)	0.743 (0.685)	−0.382 (1.100)	−0.697 (0.917)	0.769 (0.954)
	p = 0.499	p = 0.279	p = 0.729	p = 0.448	p = 0.421
**Controls**					
Gender	3.954 (0.408)	2.788 (0.345)	1.916 (0.546)	1.827 (0.469)	1.429 (0.485)
	p = 0.000	p = 0.000	p = 0.0005	p = 0.0002	p = 0.004
University	−0.482 (0.425)	−0.791 (0.360)	−0.948 (0.558)	−1.070 (0.491)	−0.491 (0.510)
	p = 0.257	p = 0.029	p = 0.090	p = 0.030	p = 0.337
Age 25-44	−4.281 (0.836)	−3.371 (0.713)	−2.503 (1.124)	−1.841 (0.958)	−3.590 (1.009)
	p = 0.00000	p = 0.00001	p = 0.027	p = 0.055	p = 0.0004
Age 45-54	−7.067 (0.878)	−6.261 (0.751)	−5.582 (1.221)	−4.037 (0.991)	−5.192 (1.062)
	p = 0.000	p = 0.000	p = 0.00001	p = 0.0001	p = 0.00001
Age 55+	−9.496 (0.802)	−8.580 (0.683)	−7.443 (1.094)	−5.567 (0.911)	−6.655 (0.962)
	p = 0.000	p = 0.000	p = 0.000	p = 0.000	p = 0.000
Observations	2,675	2,623	893	878	847
R^2	0.116	0.122	0.111	0.098	0.097
Adjusted R^2	0.112	0.118	0.099	0.086	0.084

Table SM9: Effects of treatment on precision of vote share expectations

	Dependent variable:				
	Emmanuel Macron	Marine Le Pen	Jean-Luc Mélenchon	Éric Zemmour	Valérie Pécresse
	(1)	(2)	(3)	(4)	(5)
Constant	18.499 (0.885)	15.749 (0.757)	14.346 (1.221)	12.388 (0.998)	12.485 (1.065)
	p = 0.000	p = 0.000	p = 0.000	p = 0.000	p = 0.000
**Treatment**					
Vote share	−0.915 (0.403)	−0.365 (0.342)	−0.353 (0.535)	−1.077 (0.463)	0.396 (0.479)
	p = 0.024	p = 0.286	p = 0.509	p = 0.021	p = 0.410
Probability	1.167 (0.404)	1.066 (0.342)	0.296 (0.535)	0.077 (0.462)	0.964 (0.481)
	p = 0.004	p = 0.002	p = 0.581	p = 0.868	p = 0.046
Qualitative	−0.609 (0.404)	−0.201 (0.342)	−0.541 (0.535)	0.109 (0.464)	−0.526 (0.480)
	p = 0.132	p = 0.557	p = 0.313	p = 0.815	p = 0.275
**Controls**					
Gender	3.964 (0.407)	2.777 (0.345)	1.928 (0.544)	1.863 (0.469)	1.437 (0.485)
	p = 0.000	p = 0.000	p = 0.0005	p = 0.0001	p = 0.004
University	−0.427 (0.424)	−0.756 (0.360)	−0.942 (0.556)	−1.024 (0.490)	−0.513 (0.508)
	p = 0.314	p = 0.036	p = 0.091	p = 0.038	p = 0.314
Age 25-44	−4.283 (0.836)	−3.361 (0.713)	−2.556 (1.119)	−1.675 (0.954)	−3.548 (1.008)
	p = 0.00000	p = 0.00001	p = 0.023	p = 0.080	p = 0.0005
Age 45-54	−7.057 (0.878)	−6.241 (0.750)	−5.609 (1.217)	−3.849 (0.986)	−5.143 (1.061)
	p = 0.000	p = 0.000	p = 0.00001	p = 0.0002	p = 0.00001
Age 55+	−9.463 (0.802)	−8.566 (0.682)	−7.484 (1.091)	−5.391 (0.907)	−6.600 (0.961)
	p = 0.000	p = 0.000	p = 0.000	p = 0.000	p = 0.000
Observations	2,675	2,623	893	878	847
R^2	0.114	0.120	0.110	0.093	0.089
Adjusted R^2	0.111	0.117	0.102	0.085	0.080

Table SM10: Effects of condition on correct second round prediction

	Dependent variable:
	Second round prediction
Constant	0.392 (0.041)
	p = 0.000
**Condition**	
Vote share only	0.032 (0.034)
	p = 0.353
Probability only	0.024 (0.036)
	p = 0.497
Qualitative only	0.007 (0.035)
	p = 0.832
Vote share and probability	0.101 (0.035)
	p = 0.004
Vote share and qualitative	0.055 (0.035)
	p = 0.112
Probability and qualitative	0.051 (0.034)
	p = 0.137
Vote share, probability and qualitative	0.071 (0.035)
	p = 0.041
**Controls**	
Gender	0.019 (0.018)
	p = 0.283
University	0.050 (0.018)
	p = 0.007
Age 25-44	0.114 (0.035)
	p = 0.002
Age 45-54	0.185 (0.037)
	p = 0.00000
Age 55+	0.254 (0.034)
	p = 0.000
Observations	2,934
R^2	0.031
Adjusted R^2	0.027

Table SM11: Effects of treatment on correct second round prediction

	Dependent variable:
	Second round prediction
Constant	0.391 (0.037)
	p = 0.000
**Treatment**	
Vote share	0.044 (0.017)
	p = 0.013
Probability	0.039 (0.017)
	p = 0.027
Qualitative	0.006 (0.017)
	p = 0.716
**Controls**	
Gender	0.019 (0.018)
	p = 0.285
University	0.050 (0.018)
	p = 0.007
Age 25-44	0.113 (0.035)
	p = 0.002
Age 45-54	0.184 (0.037)
	p = 0.00000
Age 55+	0.253 (0.034)
	p = 0.000
Observations	2,934
R^2	0.030
Adjusted R^2	0.027

SM8 Main models without controls

Tables SM12-SM21 provide summaries of models equivalent to those reported in the main text, but without controlling for pre-registered demographic variables (gender, education, and age): effects of condition and treatment independent variable specifications on average vote share expectations, on accuracy of vote share expectations, on precision of vote share expectations, and on correctly predicting which candidates would reach the second round. Across these models, results are consistent in direction and magnitude with those reported in the main text and in SM7, where we adjust for pre-registered demographic variables. However, owing to the exclusion of control variables, effects are estimated with less precision (i.e. larger standard errors) such that in some cases, effects that are statistically significant in our main models are marginally non-significant here. For example, in Table SM21, the effect of probability just fails to reach statistical significance at the 5% level ($p = .051$). In SM9 below we also show that randomisation of forecast conditions was successful across the our control variable. We opt to report results from the models including the controls both because this reflects our pre-registered procedure and because the estimates in those models are more precise, owing to the inclusion of pre-treatment variables that are strongly correlated with the dependent variable (Bowers 2011).

Table SM12: Effects of condition on vote share expectations, without pre-registered controls

	Dependent variable:				
	Emmanuel Macron	Marine Le Pen	Jean-Luc Mélenchon	Éric Zemmour	Valérie Pécresse
	(1)	(2)	(3)	(4)	(5)
Constant	39.757 (0.965)	34.274 (0.950)	25.933 (1.571)	19.431 (1.602)	23.385 (1.493)
	p = 0.000	p = 0.000	p = 0.000	p = 0.000	p = 0.000
**Condition**					
Vote share only	−4.485 (1.369)	−3.151 (1.348)	−3.617 (2.163)	2.662 (2.270)	−6.019 (2.194)
	p = 0.002	p = 0.020	p = 0.095	p = 0.242	p = 0.007
Probability only	4.721 (1.419)	2.867 (1.397)	−0.220 (2.319)	4.508 (2.339)	−2.021 (2.205)
	p = 0.001	p = 0.041	p = 0.925	p = 0.055	p = 0.360
Qualitative only	−1.272 (1.379)	−0.448 (1.358)	−2.094 (2.175)	3.119 (2.361)	−3.917 (2.145)
	p = 0.357	p = 0.742	p = 0.336	p = 0.187	p = 0.069
Vote share and probability	0.928 (1.375)	1.639 (1.353)	−0.183 (2.200)	1.607 (2.257)	−1.529 (2.200)
	p = 0.500	p = 0.226	p = 0.934	p = 0.477	p = 0.488
Vote share and qualitative	−3.505 (1.374)	−3.434 (1.352)	−4.307 (2.204)	1.988 (2.270)	−6.043 (2.169)
	p = 0.011	p = 0.012	p = 0.051	p = 0.382	p = 0.006
Probability and qualitative	3.138 (1.354)	1.189 (1.333)	−1.411 (2.137)	5.236 (2.284)	−4.272 (2.136)
	p = 0.021	p = 0.373	p = 0.510	p = 0.023	p = 0.046
Vote share, probability and qualitative	2.443 (1.378)	2.282 (1.358)	1.644 (2.175)	2.022 (2.328)	−2.919 (2.174)
	p = 0.077	p = 0.093	p = 0.450	p = 0.386	p = 0.180
Observations	2,934	2,933	1,000	989	942
R^2	0.025	0.014	0.012	0.007	0.014
Adjusted R^2	0.022	0.012	0.005	0.0003	0.007

Table SM13: Effects of treatment on vote share expectations, without pre-registered controls

	Dependent variable:				
	Emmanuel Macron	Marine Le Pen	Jean-Luc Mélenchon	Éric Zemmour	Valérie Pécresse
	(1)	(2)	(3)	(4)	(5)
Constant	38.887 (0.693)	33.534 (0.682)	23.995 (1.103)	21.425 (1.161)	21.288 (1.089)
	p = 0.000	p = 0.000	p = 0.000	p = 0.000	p = 0.000
**Treatment**					
Vote share	−2.785 (0.694)	−1.562 (0.683)	−0.649 (1.087)	−1.076 (1.162)	−1.623 (1.111)
	p = 0.0001	p = 0.023	p = 0.551	p = 0.355	p = 0.145
Probability	5.086 (0.694)	3.732 (0.683)	2.511 (1.086)	1.401 (1.162)	1.205 (1.111)
	p = 0.000	p = 0.00000	p = 0.022	p = 0.229	p = 0.279
Qualitative	−0.096 (0.694)	−0.475 (0.684)	−0.548 (1.089)	0.953 (1.162)	−2.005 (1.111)
	p = 0.890	p = 0.487	p = 0.616	p = 0.413	p = 0.072
Observations	2,934	2,933	1,000	989	942
R^2	0.023	0.012	0.006	0.003	0.007
Adjusted R^2	0.022	0.011	0.003	0.00004	0.004

Table SM14: Effects of condition on accuracy of vote share expectations, without pre-registered controls

	Dependent variable:				
	Emmanuel Macron	Marine Le Pen	Jean-Luc Mélenchon	Éric Zemmour	Valérie Pécresse
	(1)	(2)	(3)	(4)	(5)
Constant	16.082 (0.828)	15.499 (0.900)	13.017 (1.443)	9.677 (1.510)	14.169 (1.413)
	p = 0.000	p = 0.000	p = 0.000	p = 0.000	p = 0.000
**Condition**					
Vote share only	−3.857 (1.175)	−2.894 (1.277)	−3.415 (1.986)	2.935 (2.139)	−5.142 (2.077)
	p = 0.002	p = 0.024	p = 0.086	p = 0.171	p = 0.014
Probability only	3.624 (1.218)	2.557 (1.323)	−0.700 (2.130)	4.671 (2.204)	−1.024 (2.087)
	p = 0.003	p = 0.054	p = 0.743	p = 0.035	p = 0.624
Qualitative only	−0.977 (1.184)	−0.664 (1.287)	−2.378 (1.997)	3.765 (2.225)	−3.489 (2.031)
	p = 0.410	p = 0.606	p = 0.234	p = 0.091	p = 0.087
Vote share and probability	1.185 (1.180)	1.518 (1.282)	0.007 (2.020)	1.967 (2.127)	−0.962 (2.082)
	p = 0.316	p = 0.237	p = 0.998	p = 0.356	p = 0.645
Vote share and qualitative	−3.486 (1.179)	−3.244 (1.281)	−4.375 (2.024)	2.571 (2.139)	−5.716 (2.053)
	p = 0.004	p = 0.012	p = 0.031	p = 0.230	p = 0.006
Probability and qualitative	2.156 (1.162)	0.772 (1.263)	−1.010 (1.963)	5.260 (2.152)	−3.460 (2.023)
	p = 0.064	p = 0.541	p = 0.608	p = 0.015	p = 0.088
Vote share, probability and qualitative	2.696 (1.183)	2.545 (1.287)	1.883 (1.997)	2.622 (2.194)	−1.928 (2.058)
	p = 0.023	p = 0.048	p = 0.346	p = 0.233	p = 0.350
Observations	2,934	2,933	1,000	989	942
R^2	0.025	0.014	0.015	0.008	0.014
Adjusted R^2	0.023	0.012	0.008	0.001	0.007

Table SM15: Effects of treatment on accuracy of vote share expectations, without pre-registered controls

	Dependent variable:				
	Emmanuel Macron	Marine Le Pen	Jean-Luc Mélenchon	Éric Zemmour	Valérie Pécresse
	(1)	(2)	(3)	(4)	(5)
Constant	15.116 (0.595)	14.627 (0.647)	10.898 (1.014)	11.779 (1.094)	12.377 (1.031)
	p = 0.000	p = 0.000	p = 0.000	p = 0.000	p = 0.000
**Treatment**					
Vote share	−2.059 (0.596)	−1.179 (0.648)	−0.442 (0.999)	−0.818 (1.096)	−1.482 (1.052)
	p = 0.001	p = 0.069	p = 0.659	p = 0.456	p = 0.160
Probability	4.466 (0.596)	3.524 (0.648)	2.669 (0.998)	1.318 (1.095)	1.652 (1.052)
	p = 0.000	p = 0.00000	p = 0.008	p = 0.230	p = 0.117
Qualitative	−0.164 (0.596)	−0.478 (0.648)	−0.467 (1.001)	1.211 (1.096)	−1.962 (1.051)
	p = 0.784	p = 0.461	p = 0.641	p = 0.270	p = 0.063
Observations	2,934	2,933	1,000	989	942
R^2	0.023	0.011	0.007	0.003	0.008
Adjusted R^2	0.022	0.010	0.004	0.0003	0.005

Table SM16: Effects of condition on accuracy of vote share expectations (relative to election result), without pre-registered controls

	Dependent variable:				
	Emmanuel Macron	Marine Le Pen	Jean-Luc Mélenchon	Éric Zemmour	Valérie Pécresse
	(1)	(2)	(3)	(4)	(5)
Constant	15.694 (0.806)	14.140 (0.843)	12.605 (1.155)	12.569 (1.580)	18.508 (1.479)
	p = 0.000	p = 0.000	p = 0.000	p = 0.000	p = 0.000
**Condition**					
Vote share only	−3.592 (1.143)	−2.354 (1.196)	−1.838 (1.590)	2.803 (2.239)	−5.981 (2.174)
	p = 0.002	p = 0.050	p = 0.248	p = 0.211	p = 0.007
Probability only	3.545 (1.185)	2.357 (1.239)	−0.833 (1.704)	4.613 (2.306)	−1.890 (2.184)
	p = 0.003	p = 0.058	p = 0.626	p = 0.046	p = 0.388
Qualitative only	−0.873 (1.152)	−0.652 (1.205)	−1.890 (1.598)	3.431 (2.328)	−3.926 (2.125)
	p = 0.449	p = 0.589	p = 0.238	p = 0.141	p = 0.066
Vote share and probability	1.257 (1.148)	1.382 (1.201)	0.290 (1.617)	1.726 (2.226)	−1.508 (2.179)
	p = 0.274	p = 0.250	p = 0.858	p = 0.439	p = 0.490
Vote share and qualitative	−3.285 (1.147)	−2.777 (1.200)	−3.418 (1.620)	2.252 (2.239)	−6.080 (2.149)
	p = 0.005	p = 0.021	p = 0.036	p = 0.315	p = 0.005
Probability and qualitative	2.040 (1.130)	0.630 (1.183)	−0.255 (1.571)	5.304 (2.252)	−4.169 (2.117)
	p = 0.072	p = 0.595	p = 0.872	p = 0.019	p = 0.050
Vote share, probability and qualitative	2.743 (1.151)	2.513 (1.205)	2.280 (1.598)	2.328 (2.296)	−2.749 (2.154)
	p = 0.018	p = 0.038	p = 0.155	p = 0.311	p = 0.203
Observations	2,934	2,933	1,000	989	942
R^2	0.024	0.013	0.016	0.008	0.014
Adjusted R^2	0.022	0.010	0.009	0.001	0.007

Table SM17: Effects of treatment on accuracy of vote share expectations (relative to election result), without pre-registered controls

	Dependent variable:				
	Emmanuel Macron	Marine Le Pen	Jean-Luc Mélenchon	Éric Zemmour	Valérie Pécresse
	(1)	(2)	(3)	(4)	(5)
Constant	14.787 (0.579)	13.361 (0.606)	10.898 (0.812)	14.621 (1.145)	16.408 (1.079)
	p = 0.000	p = 0.000	p = 0.000	p = 0.000	p = 0.000
**Treatment**					
Vote share	−1.888 (0.580)	−0.885 (0.606)	0.066 (0.800)	−0.985 (1.146)	−1.630 (1.101)
	p = 0.002	p = 0.145	p = 0.935	p = 0.391	p = 0.140
Probability	4.305 (0.580)	3.141 (0.607)	2.243 (0.799)	1.372 (1.146)	1.314 (1.101)
	p = 0.000	p = 0.00000	p = 0.006	p = 0.232	p = 0.233
Qualitative	−0.169 (0.580)	−0.448 (0.607)	−0.258 (0.801)	1.097 (1.146)	−1.996 (1.101)
	p = 0.771	p = 0.460	p = 0.748	p = 0.339	p = 0.071
Observations	2,934	2,933	1,000	989	942
R^2	0.022	0.010	0.008	0.003	0.007
Adjusted R^2	0.021	0.009	0.005	0.0002	0.004

Table SM18: Effects of condition on precision of vote share expectations, without pre-registered controls

	Dependent variable:				
	Emmanuel Macron	Marine Le Pen	Jean-Luc Mélenchon	Éric Zemmour	Valérie Pécresse
	(1)	(2)	(3)	(4)	(5)
Constant	13.875 (0.596)	10.535 (0.504)	9.569 (0.830)	9.328 (0.655)	8.963 (0.697)
	p = 0.000	p = 0.000	p = 0.000	p = 0.000	p = 0.000
**Condition**					
Vote share only	−0.924 (0.840)	0.480 (0.714)	0.106 (1.123)	−0.845 (0.936)	−0.836 (1.001)
	p = 0.272	p = 0.503	p = 0.925	p = 0.367	p = 0.404
Probability only	0.261 (0.876)	0.951 (0.747)	0.983 (1.224)	0.182 (0.974)	−1.119 (1.017)
	p = 0.766	p = 0.204	p = 0.422	p = 0.852	p = 0.272
Qualitative only	−1.729 (0.851)	0.052 (0.723)	−1.027 (1.129)	−0.590 (0.972)	−1.485 (0.979)
	p = 0.043	p = 0.944	p = 0.364	p = 0.544	p = 0.130
Vote share and probability	−0.272 (0.846)	0.895 (0.715)	−0.161 (1.158)	−1.965 (0.917)	0.684 (1.001)
	p = 0.748	p = 0.212	p = 0.890	p = 0.033	p = 0.495
Vote share and qualitative	−2.169 (0.841)	−0.847 (0.714)	−0.508 (1.140)	−1.073 (0.945)	−1.917 (0.984)
	p = 0.010	p = 0.236	p = 0.657	p = 0.257	p = 0.052
Probability and qualitative	0.910 (0.834)	1.519 (0.706)	0.291 (1.113)	0.415 (0.947)	−0.795 (0.975)
	p = 0.275	p = 0.032	p = 0.794	p = 0.662	p = 0.416
Vote share, probability and qualitative	−0.287 (0.858)	0.976 (0.727)	0.350 (1.152)	−0.499 (0.957)	0.433 (0.991)
	p = 0.738	p = 0.180	p = 0.762	p = 0.602	p = 0.663
Observations	2,675	2,623	893	878	847
R^2	0.008	0.006	0.004	0.011	0.014
Adjusted R^2	0.005	0.003	−0.004	0.003	0.006

Table SM19: Effects of treatment on precision of vote share expectations, without pre-registered controls

	Dependent variable:				
	Emmanuel Macron	Marine Le Pen	Jean-Luc Mélenchon	Éric Zemmour	Valérie Pécresse
	(1)	(2)	(3)	(4)	(5)
Constant	13.382 (0.428)	10.677 (0.363)	9.472 (0.576)	9.165 (0.478)	8.033 (0.499)
	p = 0.000	p = 0.000	p = 0.000	p = 0.000	p = 0.000
**Treatment**					
Vote share	−0.811 (0.427)	−0.274 (0.363)	−0.076 (0.562)	−1.136 (0.482)	0.406 (0.499)
	p = 0.058	p = 0.452	p = 0.894	p = 0.019	p = 0.416
Probability	1.378 (0.427)	1.183 (0.363)	0.715 (0.561)	0.100 (0.482)	0.878 (0.499)
	p = 0.002	p = 0.002	p = 0.203	p = 0.837	p = 0.079
Qualitative	−0.601 (0.427)	−0.169 (0.363)	−0.451 (0.563)	0.255 (0.482)	−0.658 (0.499)
	p = 0.160	p = 0.642	p = 0.424	p = 0.597	p = 0.188
Observations	2,675	2,623	893	878	847
R^2	0.006	0.004	0.002	0.007	0.006
Adjusted R^2	0.005	0.003	−0.001	0.003	0.003

Table SM20: Effects of condition on correct second round prediction, without pre-registered controls

	Dependent variable:
	Second round prediction
Constant	0.609 (0.025)
	p = 0.000
**Condition**	
Vote share only	0.027 (0.035)
	p = 0.441
Probability only	0.016 (0.036)
	p = 0.653
Qualitative only	−0.001 (0.035)
	p = 0.985
Vote share and probability	0.089 (0.035)
	p = 0.012
Vote share and qualitative	0.054 (0.035)
	p = 0.120
Probability and qualitative	0.045 (0.034)
	p = 0.190
Vote share, probability and qualitative	0.066 (0.035)
	p = 0.059
Observations	2,934
R^2	0.004
Adjusted R^2	0.002

Table SM21: Effects of treatment on correct second round prediction, without pre-registered controls

	Dependent variable:
	Second round prediction
Constant	0.604 (0.018)
	p = 0.000
**Treatment**	
Vote share	0.043 (0.018)
	p = 0.014
Probability	0.035 (0.018)
	p = 0.051
Qualitative	0.008 (0.018)
	p = 0.644
Observations	2,934
R^2	0.003
Adjusted R^2	0.002

SM9 Randomisation Check

Tables SM22-SM24 display the observed and expected frequencies of each level of our demographic control variables across the possible forecast conditions, and the computed χ-squared statistic with its p-value. In no case do the observed frequencies of groups in each condition differ significantly from the expected frequencies, indicating that randomisation was successful.

Table SM22: Contingency table for gender and experimental forecast condition

	Men		Women	
Condition	Observed	Expected	Observed	Expected
1. Pure control	171	172.4489	208	206.5511
2. Vote share only	176	170.1738	198	203.8262
3. Probability only	149	148.3333	177	177.6667
4. Qualitative only	173	165.1687	190	197.8313
5. Vote share and probability	171	167.4438	197	200.5562
6. Vote share and qualitative	159	167.8988	210	201.1012
7. Probability and qualitative	176	177.9090	215	213.0910
8. Vote share, probability and qualitative	160	165.6237	204	198.3763

Chi Squared = 2.467, p = 0.93

Table SM23: Contingency table for education level and experimental forecast condition

	No university		University	
Condition	Observed	Expected	Observed	Expected
1. Pure control	223	230.0610	156	148.9390
2. Vote share only	216	227.0259	158	146.9741
3. Probability only	205	197.8889	121	128.1111
4. Qualitative only	228	220.3487	135	142.6513
5. Vote share and probability	229	223.3838	139	144.6162
6. Vote share and qualitative	229	223.9908	140	145.0092
7. Probability and qualitative	225	237.3453	166	153.6547
8. Vote share, probability and qualitative	226	220.9557	138	143.0443

Chi Squared = 5.812, p = 0.562

Table SM24: Contingency table for age group and experimental forecast condition

	Under 25		25-44		45-54		55+	
Condition	Observed	Expected	Observed	Expected	Observed	Expected	Observed	Expected
1. Pure control	33	30.61452	93	104.37355	59	69.10873	194	174.9032
2. Vote share only	31	30.21063	104	102.99659	68	68.19700	171	172.5958
3. Probability only	26	26.33333	92	89.77778	63	59.44444	145	150.4444
4. Qualitative only	28	29.32209	105	99.96728	67	66.19121	163	167.5194
5. Vote share and probability	36	29.72597	106	101.34424	67	67.10293	159	169.8269
6. Vote share and qualitative	27	29.80675	93	101.61963	67	67.28528	182	170.2883
7. Probability and qualitative	34	31.58384	104	107.67825	79	71.29686	174	180.4410
8. Vote share, probability and qualitative	22	29.40286	111	100.24267	65	66.37355	166	167.9809

Chi Squared = 14.422, p = 0.851

SM10 Effect Heterogeneity

SM10.1 Candidate Preferences

The effects of forecast formats may vary across different groups of voters. A first potential source of such heterogeneity is voters' preferences over the candidates. Voters tend to over-estimate the electoral chances of parties or candidates that they would like to see win the election—a phenomenon known as 'wishful thinking' (Babad 1997, Ganser & Riordan 2015, Hayes Jr 1936, Lazarsfeld et al. 1968, Meffert et al. 2011, Mongrain 2021, Searles et al. 2018, Stiers & Dassonneville 2018). Wishful thinking is considered to be a well-established pattern (see, e.g. Searles et al. 2018), despite the fact that little evidence demonstrates a causal influence of voters' preferences on their expectations, as opposed to a mere correlation between the two (Krizan & Windschitl 2007).

More contentious why wishful thinking occurs. Wishful thinking could be a purely cognitive phenomenon that will attenuate in the face of evidence (McAllister & Studlar 1991, Skalaban 1988), but scholars have also found it natural to treat wishful thinking as a particular form of 'partisan motivated reasoning' (Krizan & Windschitl 2007, 96) which will tend to lead voters' expectations to diverge as they interpret new information in ways that are congenial to their preferences (Druckman 2014, Lodge & Taber 2013). These conflicting possibilities raise the question of whether voters' responsiveness to forecast information varies in accordance with their preferences over the candidates in the election.

Table SM25: Variation in effects of forecast treatments on vote share expectations by level of support for the candidate.

	Dependent variable:				
	Emmanuel Macron	Marine Le Pen	Jean-Luc Mélenchon	Éric Zemmour	Valérie Pécresse
	(1)	(2)	(3)	(4)	(5)
Constant	36.658 (1.718)	32.725 (1.627)	20.310 (2.683)	21.428 (2.550)	23.963 (3.019)
	p = 0.000	p = 0.000	p = 0.000	p = 0.000	p = 0.000
**Moderator**					
Feeling thermometer	2.323 (0.324)	2.458 (0.288)	3.426 (0.496)	2.725 (0.546)	1.864 (0.637)
	p = 0.000	p = 0.000	p = 0.000	p = 0.00000	p = 0.004
**Treatment**					
Vote share	−1.221 (1.225)	0.213 (1.161)	3.389 (1.818)	−0.447 (1.715)	−2.830 (2.124)
	p = 0.319	p = 0.855	p = 0.063	p = 0.795	p = 0.184
Probability	3.659 (1.226)	3.829 (1.162)	−3.340 (1.826)	0.700 (1.711)	1.827 (2.127)
	p = 0.003	p = 0.001	p = 0.068	p = 0.683	p = 0.391
Qualitative	−0.591 (1.226)	−2.184 (1.162)	1.853 (1.825)	−0.666 (1.708)	−1.594 (2.135)
	p = 0.631	p = 0.061	p = 0.311	p = 0.697	p = 0.456
**Controls**					
Gender	6.358 (0.647)	4.836 (0.628)	3.071 (0.983)	3.740 (1.062)	1.258 (1.078)
	p = 0.000	p = 0.000	p = 0.002	p = 0.0005	p = 0.244
University	−5.493 (0.679)	−6.404 (0.663)	−3.269 (1.016)	−5.808 (1.109)	−4.173 (1.133)
	p = 0.000	p = 0.000	p = 0.002	p = 0.00000	p = 0.0003
Age 25-44	−2.065 (1.285)	−3.372 (1.247)	−4.956 (1.920)	−1.072 (2.063)	−3.583 (2.203)
	p = 0.109	p = 0.007	p = 0.010	p = 0.604	p = 0.105
Age 45-54	−4.637 (1.360)	−6.384 (1.323)	−6.361 (2.094)	−6.921 (2.149)	−5.300 (2.312)
	p = 0.001	p = 0.00001	p = 0.003	p = 0.002	p = 0.023
Age 55+	−10.991 (1.237)	−11.488 (1.201)	−10.115 (1.892)	−11.333 (1.964)	−11.590 (2.111)
	p = 0.000	p = 0.000	p = 0.00000	p = 0.000	p = 0.00000
**Interaction**					
Feeling:Vote share	−0.463 (0.323)	−0.571 (0.285)	−1.590 (0.487)	0.087 (0.527)	0.492 (0.637)
	p = 0.153	p = 0.046	p = 0.002	p = 0.870	p = 0.440
Feeling:Probability	0.372 (0.324)	−0.185 (0.285)	1.650 (0.488)	−0.012 (0.525)	−0.206 (0.640)
	p = 0.251	p = 0.517	p = 0.001	p = 0.983	p = 0.748
Feeling:Qualitative	0.145 (0.323)	0.493 (0.285)	−0.500 (0.488)	0.518 (0.522)	−0.005 (0.640)
	p = 0.655	p = 0.084	p = 0.306	p = 0.322	p = 0.995
Observations	2,932	2,932	1,000	988	942
R^2	0.168	0.182	0.226	0.195	0.104
Adjusted R^2	0.165	0.179	0.217	0.185	0.092

Table SM25 reports the results of models in which the effect of each treatment variable on vote share expectations for a given candidate is interacted with voters' feelings towards that candidate, reported on a seven-point scale. The results reveal, firstly, a robust pattern consistent with overall wishful thinking: respondents who like a candidate report significantly and substantially higher expectations for that candidate. There is little evidence that these preferences moderate responsiveness to the forecast treatments, however, except for Jean-Luc Mélenchon.[3]

To unpack these interaction effects, Figure SM3 displays the effect (and its 95% confidence interval) of the probabilistic and vote share forecast treatments on expectations for Jean-Luc Mélenchon's vote share at each level of feeling towards the candidate. Whereas, for those who most dislike Mélenchon, the effect of the probabilistic forecast treatment is weakly negative, those who are more supportive of him interpret his 10% probability of getting into the second round as corresponding to a significantly higher vote share. However, the opposite is true of the vote share treatment. The effect of the vote share treatment is weakly positive for those who least like Jean-Luc Mélenchon, raising their expectations of his vote share. But the more a respondent likes the candidate, the more negative this effect becomes. As Table SM25 clearly indicates, these more supportive respondents have much higher expectations, net of treatment. For these respondents, the 15% vote share reported in the forecast is the most surprising, causing them to revise their expectations downwards significantly.

As noted in the main text, Mélenchon is the candidate for whom our forecast was most out-of-step with the eventual election result and the current polling when many of our respondents took our survey. For such respondents, but especially for those engaging in wishful thinking about Jean-Luc Mélenchon's chances, his forecast vote share would have been surprisingly low. Meanwhile, his 10/100 probability reported in our probabilistic forecast may be sufficiently high to allow wishful thinking to affect how voters interpret the information.

[3] As we show in section SM18, the significant interaction effects for Jean-Luc Mélenchon remain significant when applying the pre-registered Benjamini-Hochberg procedure. The significant interaction effects for Marine Le Pen are not robust to this adjustment for false discovery.

Figure SM3: Interaction effects of probabilistic and vote share treatments on expectations for Jean-Luc Mélenchon, by level of candidate support.

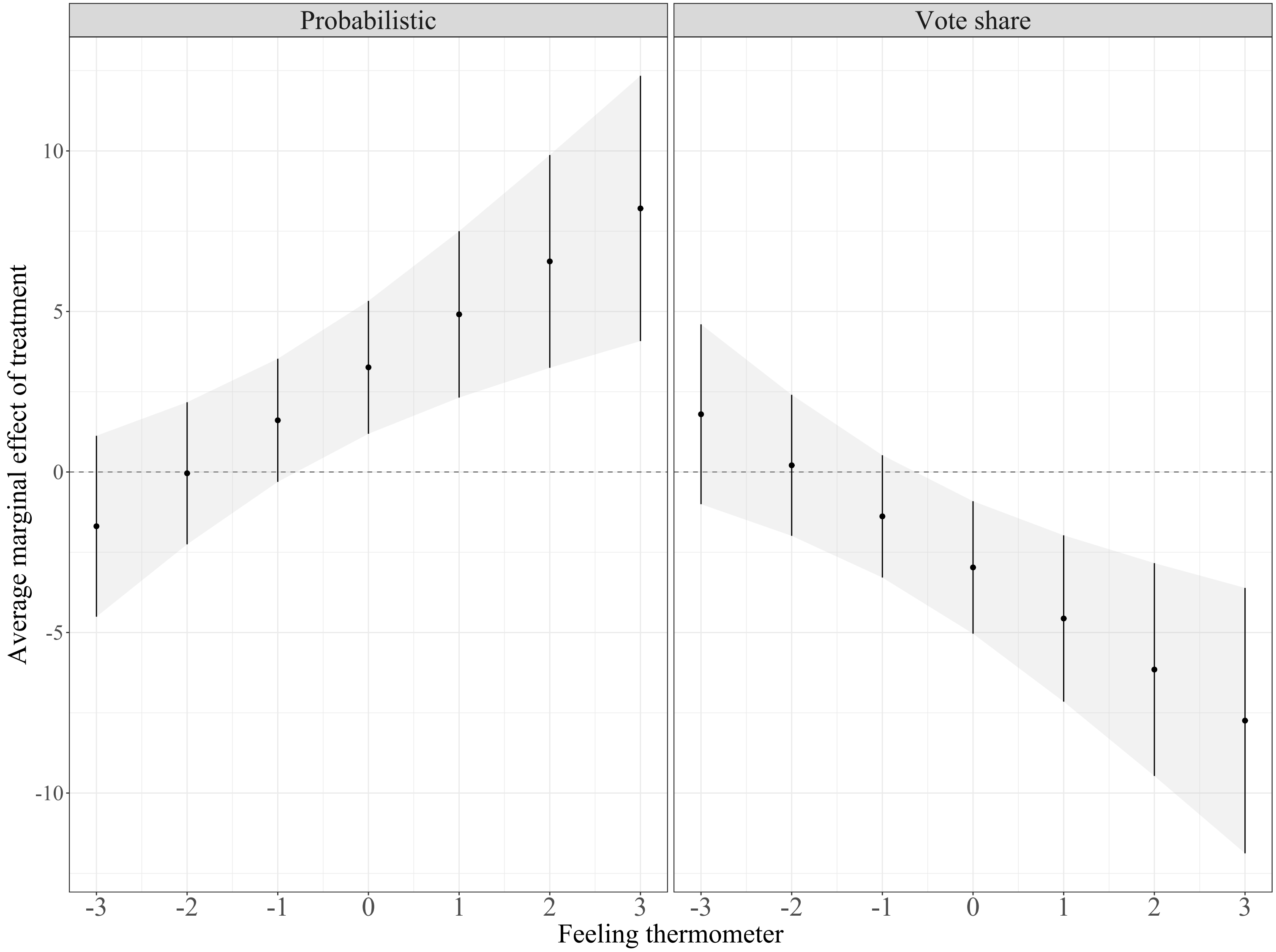

Note. Left panel shows effect of probabilistic forecast at each possible level of the Jean-Luc Mélenchon feeling thermometer. Right panel shows equivalent effect of vote share forecast.

Tables SM26 and SM27 report equivalent models to Table SM25 in which support is instead operationalised as party identification (Table SM26) and perceived ideological distance (Table SM27). Here there is little systematic evidence of any variation in treatment effects across partisans, but there is further clear evidence that party support inflates vote share expectations, captured by the main effects of each party support variable. Those who identify with a party have significantly higher expectations for its candidate's performance, and those who perceive a larger ideological distance between themselves and a given party expect that party's candidate to perform significantly worse.

Table SM26: Variation in effects of forecast treatments on vote share expectations by party identification.

	Dependent variable:				
	Emmanuel Macron	Marine Le Pen	Jean-Luc Mélenchon	Éric Zemmour	Valérie Pécresse
	(1)	(2)	(3)	(4)	(5)
Constant	43.699 (1.435)	38.518 (1.389)	31.971 (2.304)	27.227 (2.322)	29.340 (2.348)
	p = 0.000	p = 0.000	p = 0.000	p = 0.000	p = 0.000
**Moderator**					
Party ID	5.169 (1.973)	11.410 (1.786)	16.377 (3.738)	12.383 (4.485)	10.120 (3.572)
	p = 0.009	p = 0.000	p = 0.00002	p = 0.006	p = 0.005
**Treatment**					
Vote share	−2.661 (0.709)	−1.442 (0.696)	−1.019 (1.088)	−0.527 (1.154)	−1.731 (1.135)
	p = 0.0002	p = 0.039	p = 0.350	p = 0.648	p = 0.128
Probability	4.503 (0.709)	3.608 (0.697)	1.777 (1.094)	1.303 (1.152)	0.595 (1.138)
	p = 0.000	p = 0.00000	p = 0.105	p = 0.259	p = 0.602
Qualitative	0.021 (0.709)	−0.637 (0.697)	0.688 (1.089)	0.981 (1.155)	−1.590 (1.136)
	p = 0.976	p = 0.361	p = 0.528	p = 0.396	p = 0.163
**Controls**					
Gender	6.507 (0.669)	5.267 (0.644)	3.752 (1.055)	2.939 (1.129)	0.997 (1.086)
	p = 0.000	p = 0.000	p = 0.0004	p = 0.010	p = 0.359
University	−4.650 (0.699)	−6.922 (0.678)	−4.283 (1.090)	−6.581 (1.179)	−4.249 (1.140)
	p = 0.000	p = 0.000	p = 0.0001	p = 0.00000	p = 0.0003
Age 25-44	−2.901 (1.337)	−2.744 (1.286)	−6.604 (2.097)	0.141 (2.201)	−3.803 (2.222)
	p = 0.031	p = 0.033	p = 0.002	p = 0.949	p = 0.088
Age 45-54	−5.498 (1.413)	−5.554 (1.362)	−8.507 (2.270)	−5.461 (2.290)	−6.013 (2.332)
	p = 0.0002	p = 0.00005	p = 0.0002	p = 0.018	p = 0.011
Age 55+	−11.457 (1.289)	−10.663 (1.239)	−13.158 (2.052)	−10.267 (2.094)	−12.033 (2.131)
	p = 0.000	p = 0.000	p = 0.000	p = 0.00001	p = 0.00000
**Interactions**					
Party ID:Vote share	−0.467 (2.018)	−2.013 (1.746)	−4.720 (3.742)	0.584 (4.515)	0.341 (3.463)
	p = 0.817	p = 0.250	p = 0.208	p = 0.898	p = 0.922
Party ID:Probability	1.306 (2.005)	−2.107 (1.743)	0.295 (3.678)	−1.562 (4.479)	0.581 (3.470)
	p = 0.515	p = 0.227	p = 0.937	p = 0.728	p = 0.867
Party ID:Qualitative	−0.591 (2.004)	1.591 (1.745)	−9.247 (3.671)	−3.278 (4.513)	−3.565 (3.463)
	p = 0.769	p = 0.363	p = 0.012	p = 0.468	p = 0.304
Observations	2,913	2,912	992	982	936
R^2	0.115	0.145	0.109	0.105	0.091
Adjusted R^2	0.111	0.142	0.098	0.094	0.079

Table SM27: Variation in effects of forecast treatments on vote share expectations by ideological distance from party.

	Dependent variable:				
	Emmanuel Macron	Marine Le Pen	Jean-Luc Mélenchon	Éric Zemmour	Valérie Pécresse
	(1)	(2)	(3)	(4)	(5)
Constant	46.646 (1.591)	45.284 (1.535)	38.993 (2.501)	31.041 (2.608)	32.863 (2.633)
	p = 0.000	p = 0.000	p = 0.000	p = 0.000	p = 0.000
**Moderator**					
Ideological distance	−1.135 (0.293)	−1.684 (0.236)	−1.901 (0.362)	−1.189 (0.406)	−1.569 (0.483)
	p = 0.0002	p = 0.000	p = 0.00000	p = 0.004	p = 0.002
**Treatment**					
Vote share	−4.314 (0.964)	−2.545 (0.978)	−4.442 (1.640)	−0.935 (1.699)	−0.354 (1.598)
	p = 0.00001	p = 0.010	p = 0.007	p = 0.583	p = 0.825
Probability	5.232 (0.964)	2.504 (0.977)	3.147 (1.625)	1.943 (1.703)	−0.044 (1.605)
	p = 0.00000	p = 0.011	p = 0.054	p = 0.255	p = 0.979
Qualitative	0.340 (0.964)	0.405 (0.978)	−1.017 (1.623)	0.668 (1.707)	−1.880 (1.604)
	p = 0.725	p = 0.679	p = 0.532	p = 0.696	p = 0.242
**Controls**					
Gender	5.995 (0.673)	4.744 (0.644)	2.514 (1.031)	1.860 (1.121)	0.315 (1.090)
	p = 0.000	p = 0.000	p = 0.015	p = 0.098	p = 0.773
University	−4.730 (0.699)	−7.171 (0.674)	−3.886 (1.060)	−5.434 (1.166)	−3.558 (1.136)
	p = 0.000	p = 0.000	p = 0.0003	p = 0.00001	p = 0.002
Age 25-44	−2.179 (1.325)	−2.560 (1.272)	−5.287 (1.996)	1.027 (2.160)	−3.160 (2.210)
	p = 0.101	p = 0.045	p = 0.009	p = 0.635	p = 0.154
Age 45-54	−4.954 (1.404)	−4.879 (1.347)	−6.303 (2.171)	−4.588 (2.257)	−4.769 (2.318)
	p = 0.0005	p = 0.0003	p = 0.004	p = 0.043	p = 0.040
Age 55+	−10.413 (1.276)	−10.159 (1.225)	−10.361 (1.959)	−8.924 (2.059)	−10.607 (2.119)
	p = 0.000	p = 0.000	p = 0.00000	p = 0.00002	p = 0.00000
**Interactions**					
Distance:Vote share	0.558 (0.295)	0.293 (0.239)	0.763 (0.354)	0.061 (0.412)	−0.411 (0.506)
	p = 0.059	p = 0.221	p = 0.032	p = 0.882	p = 0.417
Distance:Vote share	−0.177 (0.295)	0.255 (0.239)	−0.427 (0.351)	−0.393 (0.413)	0.248 (0.511)
	p = 0.549	p = 0.287	p = 0.225	p = 0.343	p = 0.628
Distance:Qualitative	−0.201 (0.295)	−0.200 (0.239)	0.184 (0.353)	0.033 (0.415)	0.123 (0.508)
	p = 0.496	p = 0.403	p = 0.603	p = 0.938	p = 0.809
Observations	2,860	2,861	974	963	924
R^2	0.125	0.153	0.150	0.124	0.100
Adjusted R^2	0.121	0.150	0.140	0.113	0.088

Table SM28: Variation in effects of forecast treatments on vote share expectations by level of interest in politics.

	Dependent variable:				
	Emmanuel Macron	Marine Le Pen	Jean-Luc Mélenchon	Éric Zemmour	Valérie Pécresse
	(1)	(2)	(3)	(4)	(5)
Constant	50.621 (2.583)	50.773 (2.523)	40.943 (4.248)	33.036 (4.470)	42.830 (4.008)
	p = 0.000	p = 0.000	p = 0.000	p = 0.000	p = 0.000
**Moderator**					
Political interest	−2.027 (0.609)	−3.010 (0.595)	−2.079 (0.990)	−1.422 (1.061)	−3.924 (0.945)
	p = 0.001	p = 0.00000	p = 0.036	p = 0.181	p = 0.00004
**Treatment**					
Vote share	−5.371 (2.290)	−4.028 (2.237)	−2.861 (3.622)	−1.220 (3.919)	−3.176 (3.765)
	p = 0.020	p = 0.072	p = 0.430	p = 0.756	p = 0.400
Probability	9.331 (2.288)	6.468 (2.236)	3.941 (3.620)	2.959 (3.877)	1.380 (3.789)
	p = 0.00005	p = 0.004	p = 0.277	p = 0.446	p = 0.716
Qualitative	2.936 (2.293)	−1.303 (2.240)	3.726 (3.624)	3.953 (3.895)	−9.680 (3.746)
	p = 0.201	p = 0.561	p = 0.305	p = 0.311	p = 0.010
**Controls**					
Gender	5.487 (0.670)	3.978 (0.655)	2.431 (1.073)	2.046 (1.141)	0.022 (1.099)
	p = 0.000	p = 0.000	p = 0.024	p = 0.074	p = 0.985
University	−3.423 (0.700)	−6.808 (0.684)	−3.809 (1.097)	−5.493 (1.194)	−2.617 (1.152)
	p = 0.00001	p = 0.000	p = 0.001	p = 0.00001	p = 0.024
Age 25-44	−2.379 (1.311)	−2.296 (1.280)	−6.088 (2.056)	−0.080 (2.188)	−3.561 (2.213)
	p = 0.070	p = 0.074	p = 0.004	p = 0.971	p = 0.108
Age 45-54	−4.158 (1.393)	−3.559 (1.361)	−8.120 (2.235)	−5.175 (2.288)	−4.682 (2.333)
	p = 0.003	p = 0.009	p = 0.0003	p = 0.024	p = 0.046
Age 55+	−8.777 (1.288)	−8.408 (1.258)	−10.906 (2.049)	−8.902 (2.122)	−9.496 (2.159)
	p = 0.000	p = 0.000	p = 0.00000	p = 0.00003	p = 0.00002
**Interactions**					
Interest:Vote share	0.610 (0.597)	0.572 (0.583)	0.360 (0.949)	0.060 (1.019)	0.474 (0.978)
	p = 0.308	p = 0.328	p = 0.705	p = 0.954	p = 0.628
Interest:Probability	−1.249 (0.597)	−0.838 (0.583)	−0.722 (0.946)	−0.456 (1.010)	−0.011 (0.983)
	p = 0.037	p = 0.151	p = 0.446	p = 0.652	p = 0.992
Interest:Qualitative	−0.901 (0.598)	0.143 (0.584)	−1.185 (0.949)	−0.962 (1.015)	2.121 (0.973)
	p = 0.132	p = 0.808	p = 0.213	p = 0.344	p = 0.030
Observations	2,934	2,933	1,000	989	942
R^2	0.132	0.136	0.110	0.101	0.094
Adjusted R^2	0.129	0.133	0.099	0.089	0.083

SM10.2 Political Interest

A second potential source of variation in voters' responsiveness to forecast information is their level of political interest. Those who are more interested in politics are more likely to be exposed to polls and forecasts regularly in their daily lives (Zerback et al. 2021). For these people, the information conveyed by a forecast is less novel, and their expectations are likely already to be closer to reality owing to their greater familiarity with the electoral context. The highly politically interested may therefore be less responsive to our forecast treatments.

Table SM28 assesses this claim by reporting the results of models in which the effect of each treatment variable on vote share expectations for a given candidate is interacted with a respondent's level of political interest. Firstly, except in the case of Éric Zemmour, we find clear and consistent evidence that more political interested respondents, all else being equal, hold significantly lower expectations. That is—given the general tendency to vastly over-estimate each candidate's vote share—more politically interested individuals hold significantly more realistic vote share expectations. There is little evidence that this makes them less responsive to our forecast treatments. In two cases we observe statistically significant interaction effects. First, probabilistic forecasts raise expectations of Macron's vote share to a lesser extent among politically interested voters. Second, vote share forecasts decrease Valérie Pécresse's expected vote share to a lesser extent among politically interested voters. Neither effect holds up, however, when applying a preregistered Benjamini-Hochberg procedure.

Table SM29: Variation in effects of forecast treatments on vote share expectations by level of anti-expert sentiment.

	Dependent variable:				
	Emmanuel Macron	Marine Le Pen	Jean-Luc Mélenchon	Éric Zemmour	Valérie Pécresse
	(1)	(2)	(3)	(4)	(5)
Constant	45.395 (3.104)	27.880 (2.991)	32.557 (4.822)	21.162 (5.167)	13.730 (5.084)
	p = 0.000	p = 0.000	p = 0.000	p = 0.00005	p = 0.008
**Moderator**					
Anti-expert	−0.395 (0.909)	4.252 (0.876)	0.500 (1.423)	2.422 (1.537)	5.124 (1.452)
	p = 0.664	p = 0.00001	p = 0.726	p = 0.116	p = 0.0005
**Treatment**					
Vote share	−6.088 (2.739)	−3.675 (2.639)	−6.473 (4.271)	−5.064 (4.665)	3.817 (4.367)
	p = 0.027	p = 0.164	p = 0.130	p = 0.278	p = 0.383
Probability	2.726 (2.746)	2.069 (2.646)	−7.886 (4.260)	−4.747 (4.666)	3.690 (4.396)
	p = 0.321	p = 0.435	p = 0.065	p = 0.310	p = 0.402
Qualitative	1.610 (2.745)	3.670 (2.645)	4.497 (4.303)	−0.275 (4.653)	2.180 (4.385)
	p = 0.558	p = 0.166	p = 0.297	p = 0.953	p = 0.620
**Controls**					
Gender	6.507 (0.673)	5.539 (0.648)	3.878 (1.067)	3.180 (1.121)	1.281 (1.087)
	p = 0.000	p = 0.000	p = 0.0003	p = 0.005	p = 0.239
University	−4.490 (0.704)	−7.330 (0.679)	−4.590 (1.101)	−5.879 (1.171)	−2.958 (1.152)
	p = 0.000	p = 0.000	p = 0.00004	p = 0.00000	p = 0.011
Age 25-44	−2.701 (1.337)	−3.300 (1.288)	−6.820 (2.099)	−0.763 (2.182)	−4.122 (2.220)
	p = 0.044	p = 0.011	p = 0.002	p = 0.727	p = 0.064
Age 45-54	−5.329 (1.413)	−5.426 (1.362)	−9.214 (2.280)	−6.315 (2.266)	−6.157 (2.333)
	p = 0.0002	p = 0.0001	p = 0.0001	p = 0.006	p = 0.009
Age 55+	−11.098 (1.287)	−11.521 (1.241)	−13.470 (2.051)	−11.204 (2.078)	−11.803 (2.125)
	p = 0.000	p = 0.000	p = 0.000	p = 0.00000	p = 0.00000
**Interactions**					
Anti-expert:Vote share	1.045 (0.874)	0.673 (0.842)	1.687 (1.353)	1.471 (1.491)	−1.725 (1.402)
	p = 0.232	p = 0.425	p = 0.213	p = 0.324	p = 0.219
Anti-expert:Probability	0.668 (0.876)	0.423 (0.844)	3.048 (1.347)	1.971 (1.492)	−0.812 (1.411)
	p = 0.446	p = 0.617	p = 0.024	p = 0.187	p = 0.565
Anti-expert:Qualitative	−0.576 (0.876)	−1.347 (0.844)	−1.606 (1.363)	0.308 (1.488)	−1.241 (1.407)
	p = 0.511	p = 0.111	p = 0.240	p = 0.836	p = 0.379
Observations	2,931	2,930	998	988	942
R^2	0.108	0.135	0.091	0.118	0.088
Adjusted R^2	0.104	0.131	0.080	0.107	0.076

SM10.3 Anti-expert Sentiment/Self-efficacy

Finally, the expectations of voters who are explicitly less trusting of expert knowledge, relative to their own opinions, may be less responsive to forecasts. Those who prefer to rely on their own opinions over the opinions of experts may pay less attention to the latter in forming their expectations, preferring to rely on their own predictions or other information sources.

Table SM29 assesses this possibility by interacting the effect of forecast treatments with levels of anti-expert sentiment. Once again, there is minimal evidence of any moderation. Only one interaction effect is statistically significant. suggesting that the negative effect of the probabilistic forecast on expectations for Jean-Luc Mélenchon is weaker amongst those higher in anti-expert sentiment. However, this effect is no longer significant when we apply the Benjamini-Hochberg procedure. Overall, anti-expert sentiment is associated with significantly higher expectations for both Marine Le Pen and Valérie Pécresse.

SM11 Treatment interactions

In our 'treatment' specification of our main independent variable, respondents receive the value 1 if they saw a given forecast format, and 0 if they did not. This produces three dummy variables—one for each forecast format—which we include together in the same model. Doing this assumes that the forecasts can have an additive effect on expectations, and do not interact with each other. For example, we implicitly assume in these models that if both vote share forecasts and probability forecasts individually improved the accuracy of expectations, then the effect of both forecasts presented together would also be positive, and larger. This assumption does tally with many of the results in our condition specification. For example, vote share forecasts and probability forecasts both have positive effects on ability to predict the winner, and the condition where both are combined significantly improves expectations relative to the control group. Also, the negative effect of probability forecasts appears to cancel out the positive effect of vote share forecasts on accuracy, which is also consistent with an additive effect.

Nonetheless, in Tables SM30-SM32 we assess whether the overall effects of each forecast format interact. For example, does the effect of the vote share forecast change when a probability forecast is also provided? Or perhaps probability forecasts do not help people to predict the winner if a vote share forecast is also provided—is it only in the absence of the latter that a probability forecast helps?

We find that the interactions between treatments are null almost across the board. Only in a couple of isolated cases do these interaction effects reach statistical significance, with no consistent patterns emerging across candidates.

These results appear to justify our inclusion of the implicitly additive treatment specification.

Table SM30: Effects of treatment on vote share expectations, with interactions between treatments

	Dependent variable:				
	Emmanuel Macron	Marine Le Pen	Jean-Luc Mélenchon	Éric Zemmour	Valérie Pécresse
	(1)	(2)	(3)	(4)	(5)
Constant	45.243 (1.556)	41.724 (1.523)	35.993 (2.511)	26.876 (2.601)	31.839 (2.534)
	p = 0.000	p = 0.000	p = 0.000	p = 0.000	p = 0.000
**Treatment**					
Vote share	−4.693 (1.310)	−3.352 (1.283)	−4.124 (2.092)	2.056 (2.187)	−5.676 (2.148)
	p = 0.0004	p = 0.010	p = 0.050	p = 0.348	p = 0.009
Probability	4.129 (1.359)	2.120 (1.330)	−1.434 (2.248)	3.543 (2.252)	−2.155 (2.148)
	p = 0.003	p = 0.112	p = 0.524	p = 0.116	p = 0.317
Qualitative	−1.716 (1.321)	−1.068 (1.293)	−1.901 (2.108)	1.587 (2.275)	−4.183 (2.093)
	p = 0.195	p = 0.410	p = 0.368	p = 0.486	p = 0.046
**Controls**					
Gender	6.423 (0.670)	5.052 (0.656)	3.633 (1.069)	2.716 (1.133)	0.890 (1.091)
	p = 0.000	p = 0.000	p = 0.001	p = 0.017	p = 0.416
University	−4.496 (0.699)	−7.962 (0.685)	−4.809 (1.100)	−6.253 (1.185)	−3.476 (1.151)
	p = 0.000	p = 0.000	p = 0.00002	p = 0.00000	p = 0.003
Age 25-44	−2.655 (1.329)	−2.575 (1.301)	−6.726 (2.089)	0.007 (2.208)	−3.666 (2.240)
	p = 0.046	p = 0.048	p = 0.002	p = 0.998	p = 0.103
Age 45-54	−5.302 (1.406)	−4.792 (1.377)	−8.798 (2.264)	−5.984 (2.299)	−5.947 (2.352)
	p = 0.0002	p = 0.001	p = 0.0002	p = 0.010	p = 0.012
Age 55+	−11.104 (1.280)	−10.876 (1.253)	−13.293 (2.040)	−10.298 (2.106)	−11.634 (2.148)
	p = 0.000	p = 0.000	p = 0.000	p = 0.00001	p = 0.00000
**Treatment Interactions**					
Vote share:Probability	0.792 (1.894)	2.038 (1.854)	4.145 (3.050)	−4.368 (3.130)	5.595 (3.095)
	p = 0.676	p = 0.272	p = 0.175	p = 0.164	p = 0.072
Vote share:Qualitative	2.576 (1.867)	0.565 (1.828)	1.256 (2.957)	−1.880 (3.166)	3.888 (3.045)
	p = 0.168	p = 0.758	p = 0.672	p = 0.553	p = 0.203
Probability:Qualitative	0.334 (1.889)	−0.217 (1.849)	1.114 (3.022)	−0.584 (3.212)	2.273 (3.012)
	p = 0.860	p = 0.907	p = 0.713	p = 0.856	p = 0.451
Vote share:Probability:Qualitative	0.459 (2.660)	1.533 (2.604)	1.118 (4.212)	1.072 (4.484)	−2.124 (4.333)
	p = 0.863	p = 0.557	p = 0.791	p = 0.812	p = 0.625
Observations	2,934	2,933	1,000	989	942
R^2	0.109	0.109	0.085	0.088	0.072
Adjusted R^2	0.105	0.105	0.074	0.077	0.060

Table SM31: Effects of treatment on precision of vote share expectations, with interactions between treatments

	Dependent variable:				
	Emmanuel Macron	Marine Le Pen	Jean-Luc Mélenchon	Éric Zemmour	Valérie Pécresse
	(1)	(2)	(3)	(4)	(5)
Constant	19.063 (0.973)	15.720 (0.831)	14.300 (1.340)	12.912 (1.113)	13.524 (1.157)
	p = 0.000	p = 0.000	p = 0.000	p = 0.000	p = 0.000
**Treatment**					
Vote share	−0.983 (0.794)	0.344 (0.672)	−0.025 (1.068)	−1.027 (0.899)	−0.735 (0.967)
	p = 0.216	p = 0.610	p = 0.982	p = 0.254	p = 0.448
Probability	0.041 (0.828)	0.721 (0.704)	0.679 (1.167)	−0.012 (0.934)	−1.004 (0.977)
	p = 0.961	p = 0.306	p = 0.561	p = 0.990	p = 0.305
Qualitative	−1.823 (0.805)	−0.197 (0.680)	−0.898 (1.075)	−1.115 (0.933)	−1.615 (0.944)
	p = 0.024	p = 0.773	p = 0.405	p = 0.233	p = 0.088
**Controls**					
Gender	3.954 (0.408)	2.788 (0.345)	1.916 (0.546)	1.827 (0.469)	1.429 (0.485)
	p = 0.000	p = 0.000	p = 0.0005	p = 0.0002	p = 0.004
University	−0.482 (0.425)	−0.791 (0.360)	−0.948 (0.558)	−1.070 (0.491)	−0.491 (0.510)
	p = 0.257	p = 0.029	p = 0.090	p = 0.030	p = 0.337
Age 25-44	−4.281 (0.836)	−3.371 (0.713)	−2.503 (1.124)	−1.841 (0.958)	−3.590 (1.009)
	p = 0.00000	p = 0.00001	p = 0.027	p = 0.055	p = 0.0004
Age 45-54	−7.067 (0.878)	−6.261 (0.751)	−5.582 (1.221)	−4.037 (0.991)	−5.192 (1.062)
	p = 0.000	p = 0.000	p = 0.00001	p = 0.0001	p = 0.00001
Age 55+	−9.496 (0.802)	−8.580 (0.683)	−7.443 (1.094)	−5.567 (0.911)	−6.655 (0.962)
	p = 0.000	p = 0.000	p = 0.000	p = 0.000	p = 0.000
**Treatment Interactions**					
Vote share:Probability	0.211 (1.149)	−0.590 (0.974)	−1.331 (1.565)	−1.136 (1.289)	2.086 (1.383)
	p = 0.855	p = 0.545	p = 0.396	p = 0.379	p = 0.132
Vote share:Qualitative	0.457 (1.130)	−1.228 (0.957)	0.114 (1.492)	0.919 (1.311)	0.452 (1.355)
	p = 0.686	p = 0.200	p = 0.940	p = 0.484	p = 0.739
Probability:Qualitative	2.461 (1.150)	0.797 (0.975)	0.104 (1.545)	1.245 (1.334)	2.020 (1.349)
	p = 0.033	p = 0.414	p = 0.947	p = 0.351	p = 0.135
Vote share:Probability:Qualitative	−0.912 (1.617)	0.895 (1.369)	0.974 (2.148)	0.429 (1.857)	−0.436 (1.920)
	p = 0.573	p = 0.514	p = 0.651	p = 0.818	p = 0.821
Observations	2,675	2,623	893	878	847
R^2	0.116	0.122	0.111	0.098	0.097
Adjusted R^2	0.112	0.118	0.099	0.086	0.084

Table SM32: Effects of treatment on predicting the second round, with interactions between treatments

	Dependent variable:
Constant	0.392 (0.041)
	p = 0.000
**Treatment**	
Vote share	0.032 (0.034)
	p = 0.353
Probability	0.024 (0.036)
	p = 0.497
Qualitative	0.007 (0.035)
	p = 0.832
**Controls**	
Gender	0.019 (0.018)
	p = 0.283
University	0.050 (0.018)
	p = 0.007
Age 25-44	0.114 (0.035)
	p = 0.002
Age 45-54	0.185 (0.037)
	p = 0.00000
Age 55+	0.254 (0.034)
	p = 0.000
**Treatment Interactions**	
Vote share:Probability	0.045 (0.050)
	p = 0.365
Vote share:Qualitative	0.015 (0.049)
	p = 0.753
Probability:Qualitative	0.019 (0.050)
	p = 0.702
Vote share:Probability:Qualitative	−0.072 (0.070)
	p = 0.301
Observations	2,934
R^2	0.031
Adjusted R^2	0.027

SM12 Effects on Vote Choice

Exposure to our forecast treatments appears to have made respondents slightly more likely to report intending to vote for Marine Le Pen. Controlling for pre-treatment vote intention, in almost every condition, the probability of reporting intending to vote for Marine Le Pen is statistically significantly higher than in the control condition. This was not the case for any of the other candidates.

To shed some further light on this effect, Figures SM5-SM7 display the changes in vote intentions from the beginning (pre-treatment) to the end (post-treatment) of our survey, among those who received the vote share (Figure SM5), probabilistic (Figure SM6), and qualitative (Figure SM7) forecasts. In every case, the overwhelming picture is of stability. Very few respondents, under any of the three treatments, defect to another candidate. This highlights that, although marginally statistically significant, any effect of forecasts on voting behaviour was extremely small and not substantively meaningful.

Figure SM4: Condition and treatment effects on vote choice.

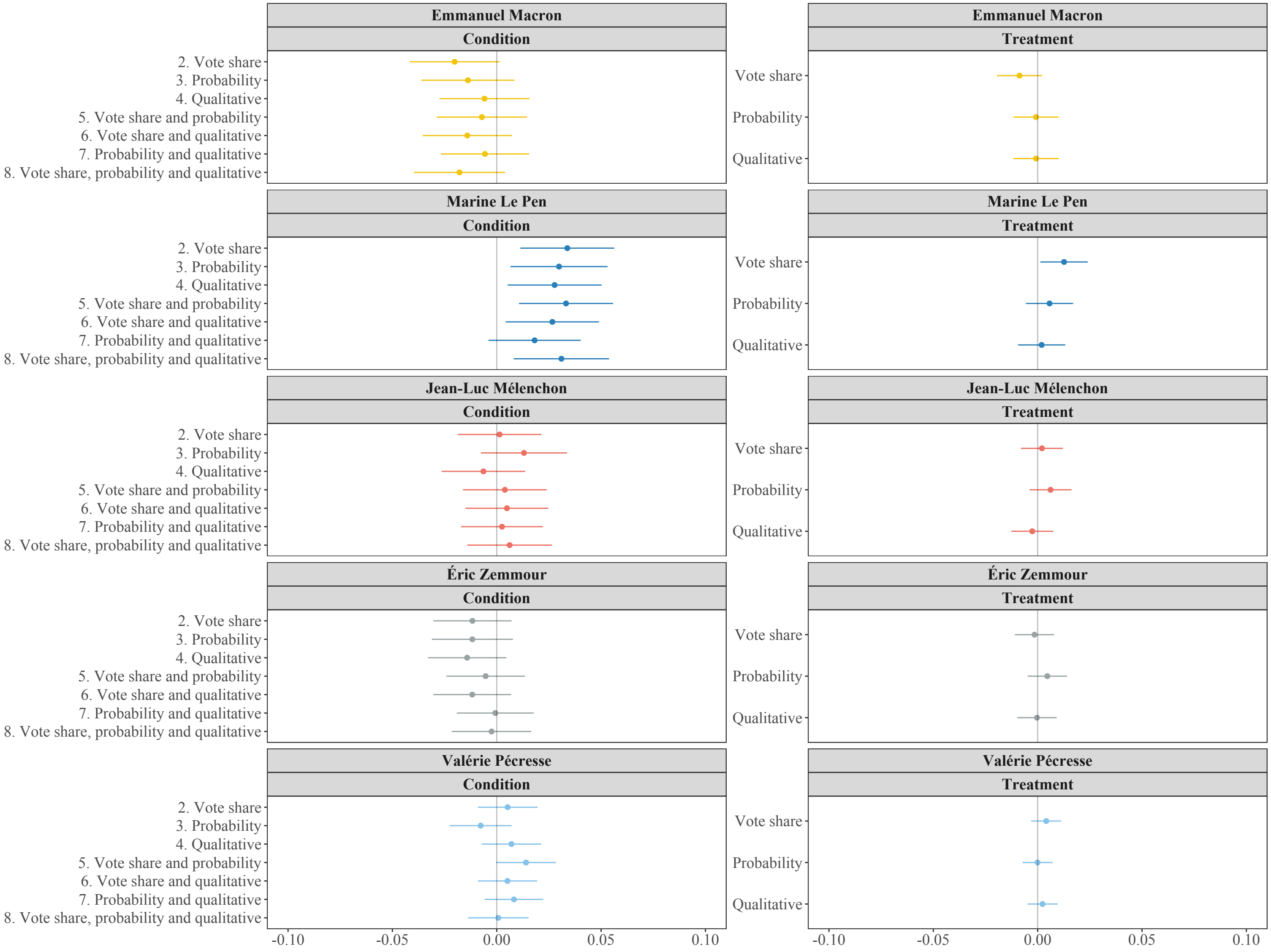

Note. Left column shows the average effect on vote choice of each condition (combination of forecast formats presented) compared to control (no forecast). Right column shows the independent average effect on vote choice of each forecast format.

Figure SM5: Changes in vote intention from pre- to post-treatment, with vote share forecast treatment.

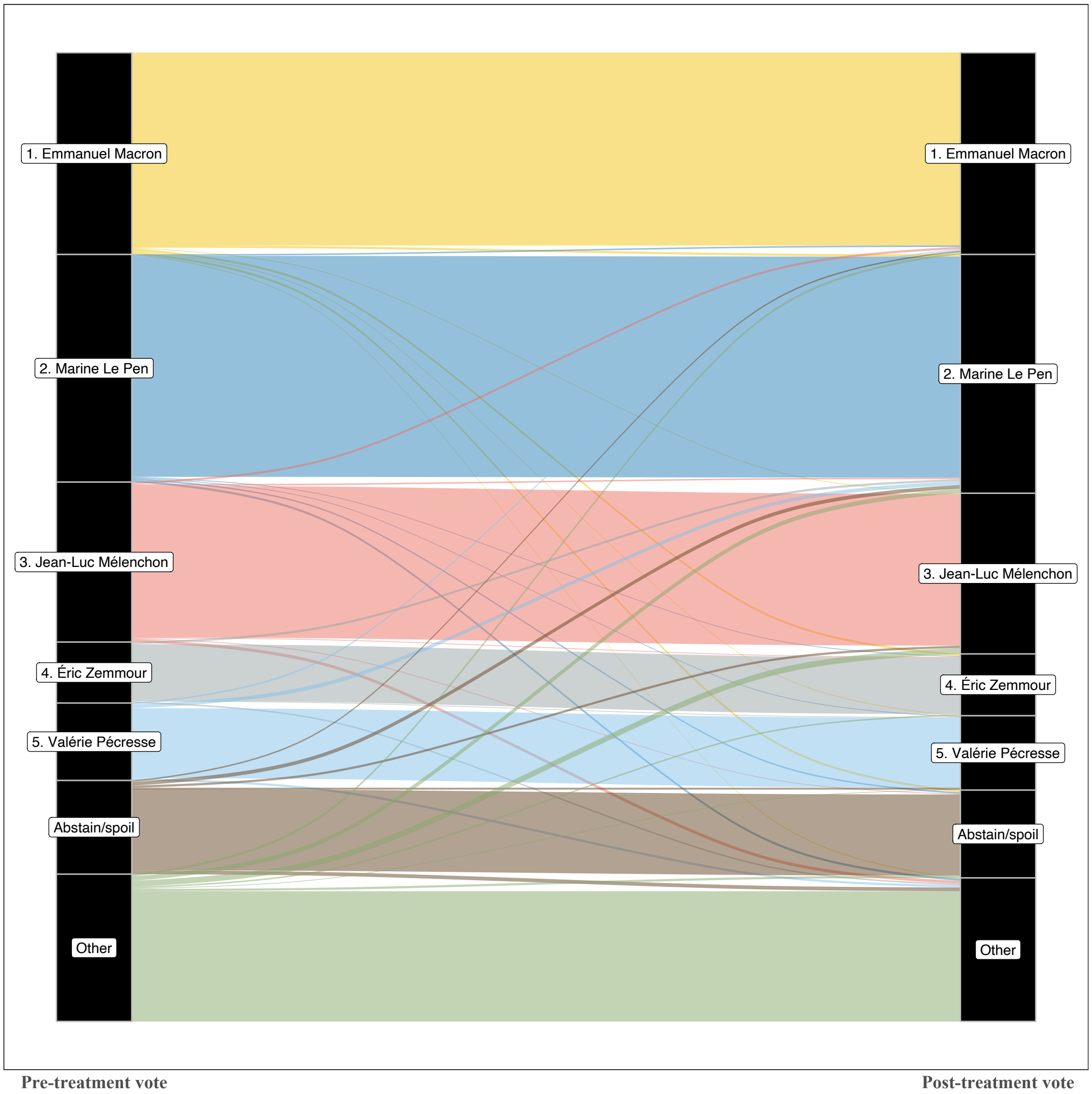

Note. Left column is proportions of respondents intending to vote for each candidate asked at the beginning of our survey, right column is proportions intending to vote for each candidate towards the end of our survey. Coloured sections show proportions of a given candidate's pre-treatment supporters who changed to support another candidate post-treatment.

Figure SM6: Changes in vote intention from pre- to post-treatment, with probabilistic forecast treatment.

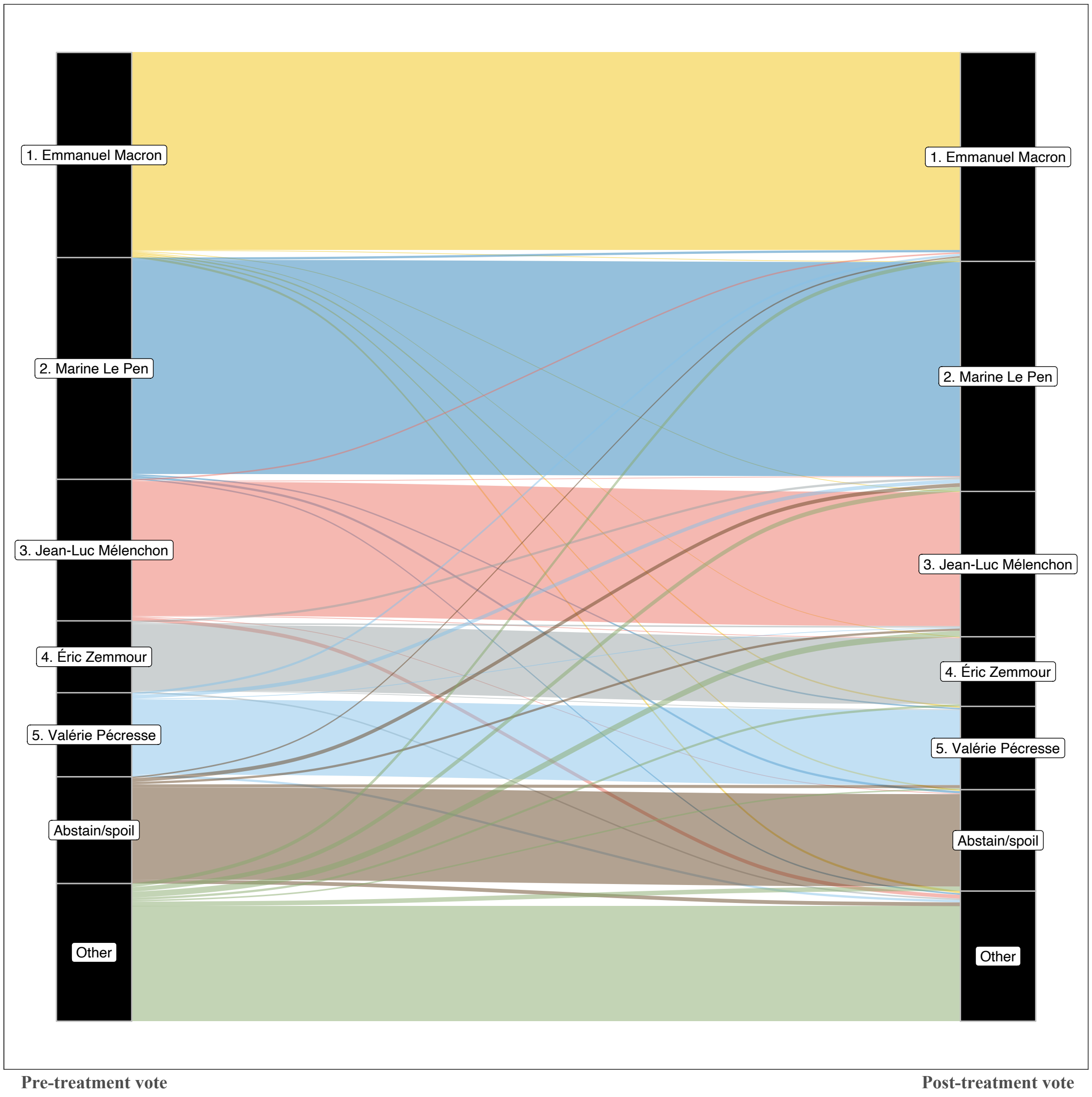

Note. Left column is proportions of respondents intending to vote for each candidate asked at the beginning of our survey, right column is proportions intending to vote for each candidate towards the end of our survey. Coloured sections show proportions of a given candidate's pre-treatment supporters who changed to support another candidate post-treatment.

Figure SM7: Changes in vote intention from pre- to post-treatment, with qualitative forecast treatment.

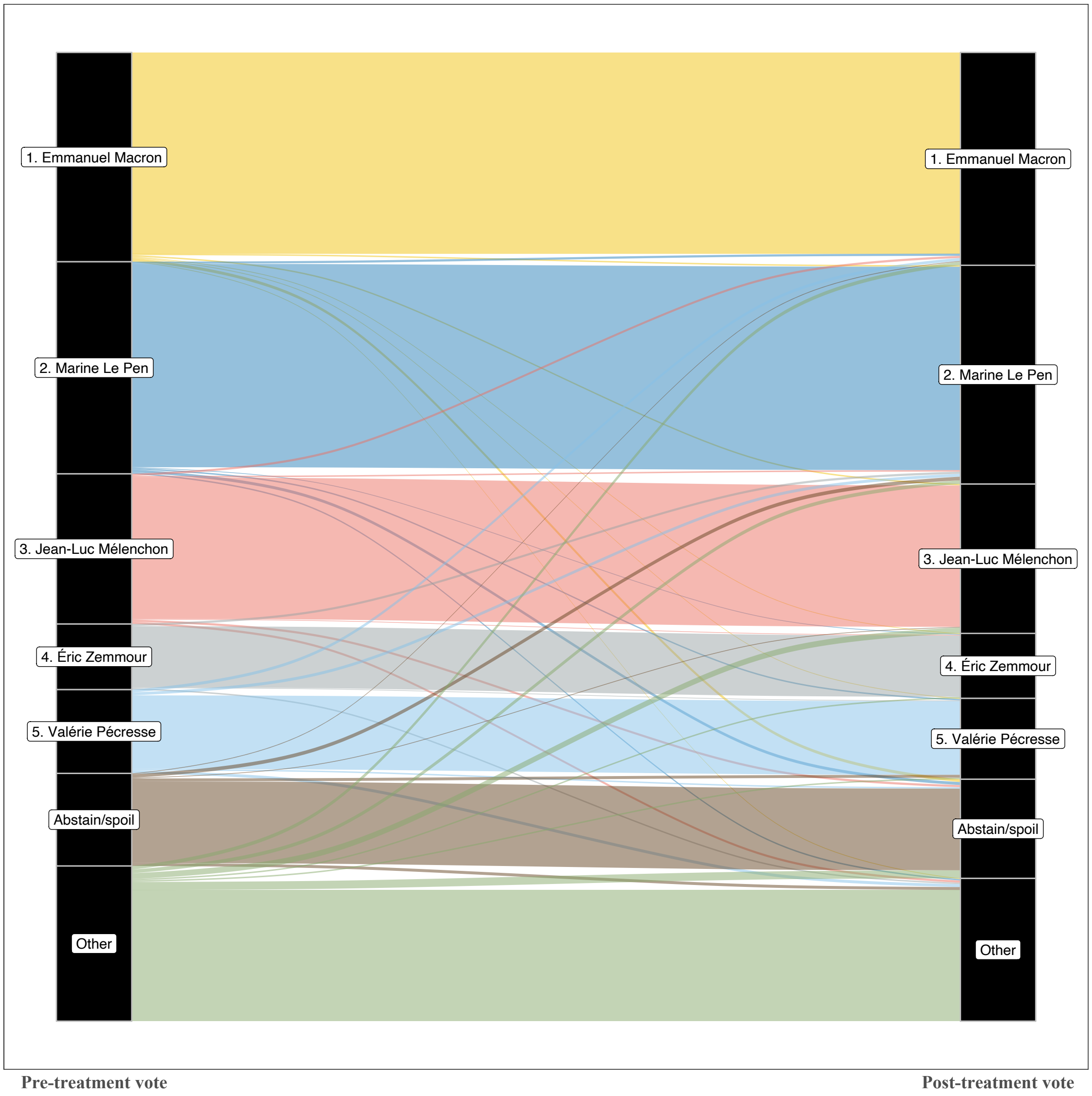

Note. Left column is proportions of respondents intending to vote for each candidate asked at the beginning of our survey, right column is proportions intending to vote for each candidate towards the end of our survey. Coloured sections show proportions of a given candidate's pre-treatment supporters who changed to support another candidate post-treatment.

SM13 Top-Two Advantage

Our forecast treatments shows a large gap between the probability of victory for the top two candidates (Emmanuel Macron and Marine Le Pen) and the probability of victory for the rest. We therefore pre-registered an analysis assessing whether the appearance of such a large gap led respondents to exaggerate the predicted difference in vote share between the top two and third place—that is, in practice, between Marine Le Pen and Jean-Luc Mélenchon. Figure SM8 demonstrates that none of the conditions or treatments discernibly had any such effect.

Figure SM8: Condition and treatment effects on difference between Le Pen and Mélenchon vote share.

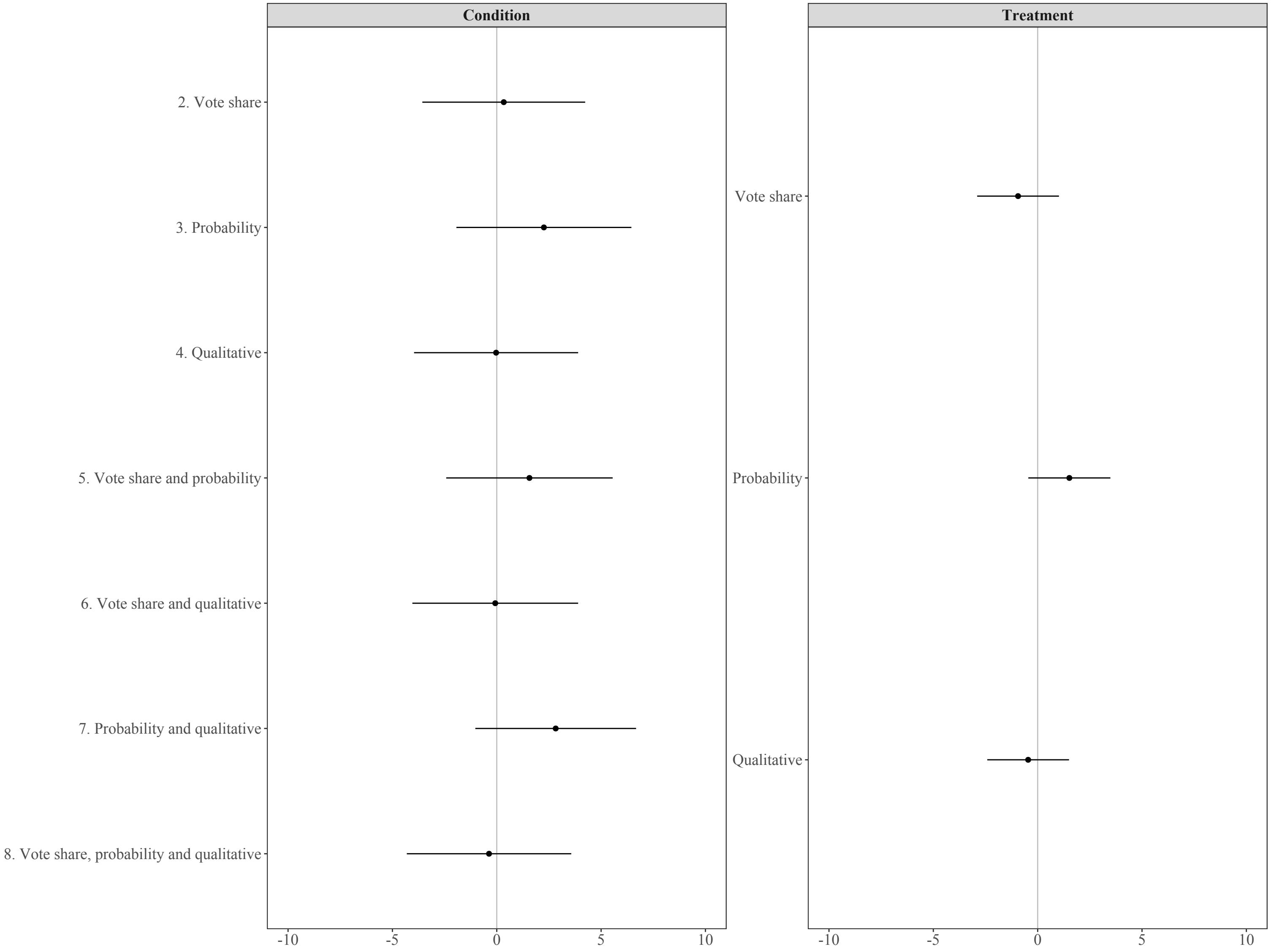

Note. Left column shows the average effect of each condition (combination of forecast formats presented) compared to control (no forecast). Right column shows the independent average effect of each forecast format.

SM14 Alternative Second Round Prediction Specification

In the main text, we reported the effects of our forecast treatments on respondents' probability of correctly predicting which two candidates would qualify for the second round of the election (Emmanuel Macron and Marine Le Pen). Figure SM9 reports, instead, the effect on the probability of predicting that each candidate will qualify for the second round. Consistent with our observation that probabilistic forecasts were no more useful in helping respondents predict the winners than vote share forecasts—but the combination of both is most useful—Figure SM9 shows that the probability of predicting either Emmanuel Macron or Marine Le Pen would be in the second round is significantly higher on average when voters receive the vote share forecast, but not so for the probabilistic forecast. These probabilities are significantly higher is when voters see both of these forecast formats, but not either in isolation.

Figure SM9: Condition and treatment effects on predicting each candidate qualifies for second round.

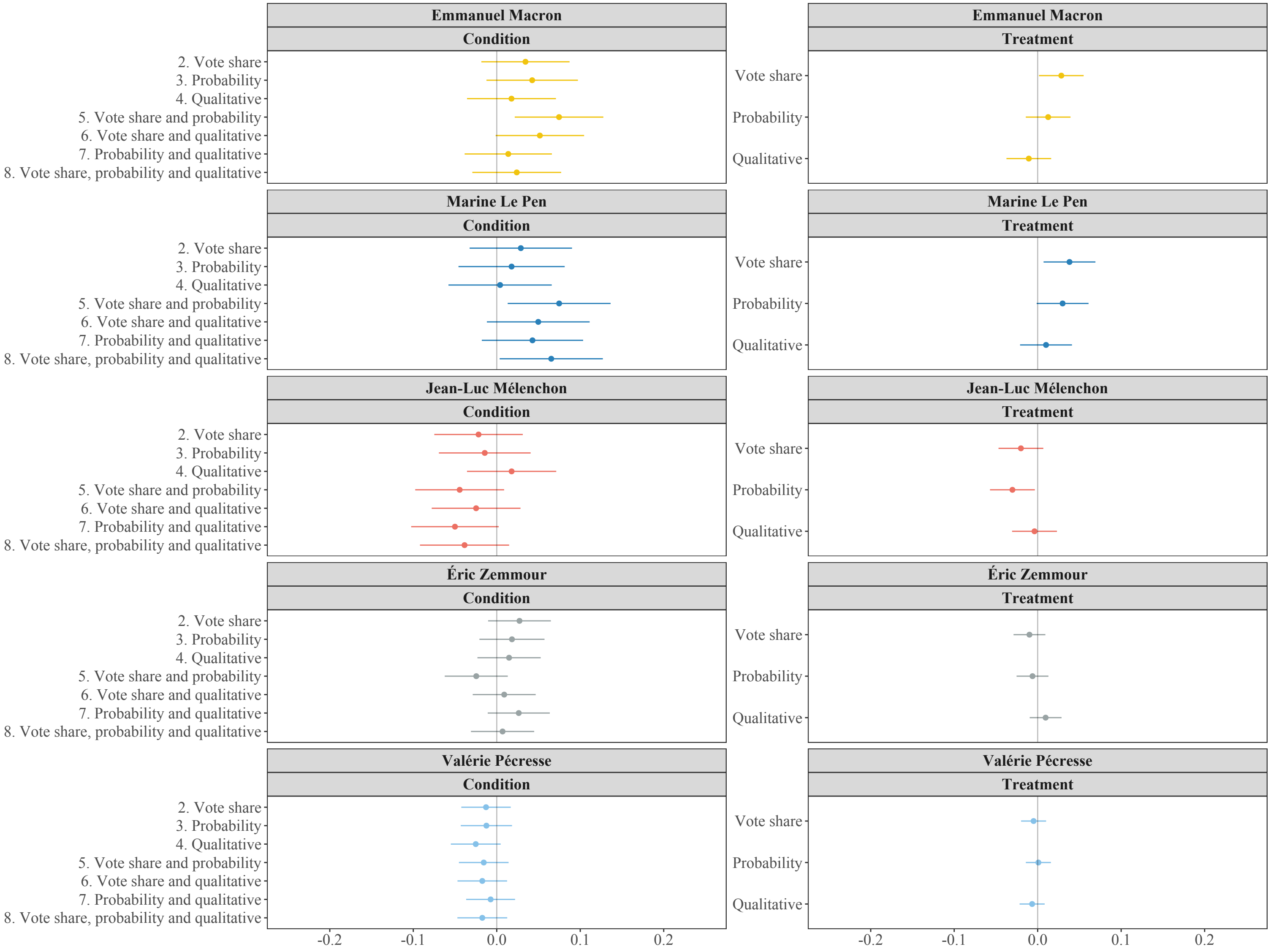

Note. Left column shows the average effect of each condition (combination of forecast formats presented) compared to control (no forecast). Right column shows the independent average effect of each forecast format.

SM15 Response Time Model for Second Round Prediction

To assess how our forecast treatments affected respondents' confidence in their predictions of which candidates would reach the second round, we measured the time it took them to answer this question. Figure SM10 shows the effects of our forecast treatments on the logged response times. Vote share forecasts, on average, appear to reduce response times, whereas probabilistic and qualitative forecasts have no significant effect. However, this effect seems to be driven largely by a reduction in response time when vote share forecasts are seen in tandem with either probabilistic or qualitative forecasts.

Figure SM10: Condition and treatment effects on logged response time for second round prediction.

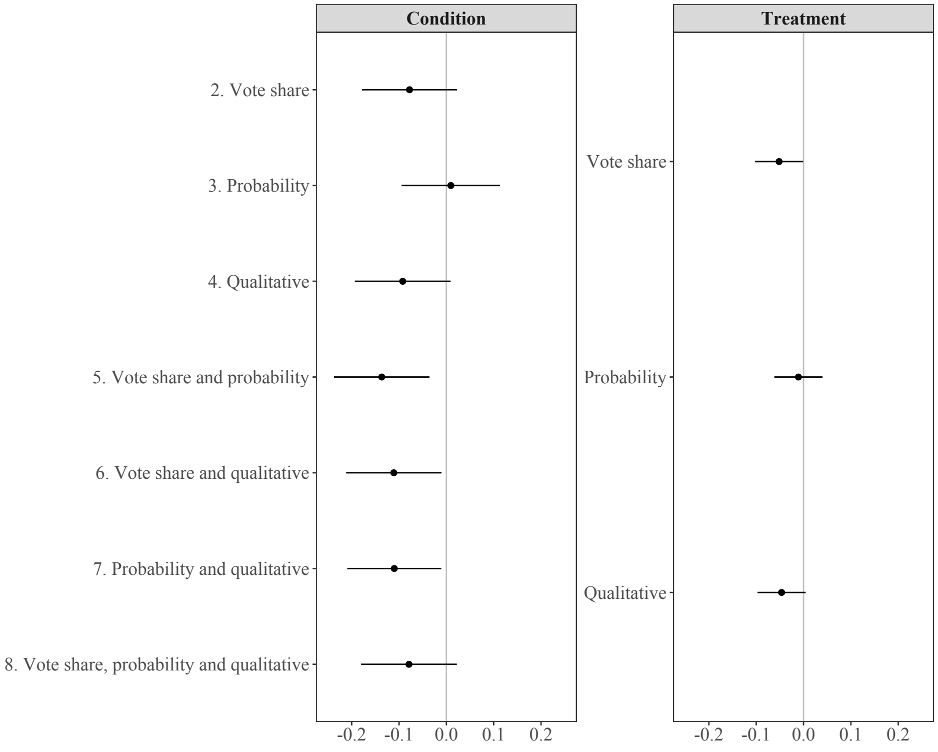

Note. Left column shows the average effect of each condition (combination of forecast formats presented) compared to control (no forecast). Right column shows the independent average effect of each forecast format.

SM16 Variation in Effects Over Time

We collected data over a period of eight days, immediately prior to the election. Over this time, the polls changed considerably, but we continued to present respondents with the same forecast, while openly telling them that it was compiled on 1st April—the first day of data collection. We predicted that the time delay between our forecast and the date on which many of our respondents completed the survey would limit how much attention those respondents paid to the forecast, reducing its effect on their expectations. Table SM33 reports the results of analyses measuring whether our treatment effects varied depending on the time/date of survey response. We split respondents into terciles based on their interview start time and interact our treatment indicators with these terciles. There is little systematic evidence of any change in treatment effects over time.

Table SM33: Variation in effects of forecast treatments on vote share expectations by survey response date.

	Dependent variable:				
	Emmanuel Macron	Marine Le Pen	Jean-Luc Mélenchon	Éric Zemmour	Valérie Pécresse
	(1)	(2)	(3)	(4)	(5)
Constant	43.362 (1.733)	39.763 (1.697)	34.778 (2.799)	29.143 (2.856)	30.159 (2.880)
	p = 0.000	p = 0.000	p = 0.000	p = 0.000	p = 0.000
**Moderator**					
Time middle tercile	1.186 (1.644)	0.794 (1.610)	−0.415 (2.668)	−2.527 (2.745)	−0.812 (2.664)
	p = 0.471	p = 0.622	p = 0.877	p = 0.358	p = 0.761
Time upper tercile	1.786 (1.633)	2.210 (1.599)	−1.899 (2.613)	−0.123 (2.762)	−0.096 (2.641)
	p = 0.275	p = 0.168	p = 0.468	p = 0.965	p = 0.971
**Treatment**					
Vote share	−3.565 (1.151)	−0.631 (1.127)	−2.232 (1.804)	−0.765 (1.948)	−2.754 (1.906)
	p = 0.002	p = 0.576	p = 0.217	p = 0.695	p = 0.149
Probability	6.547 (1.152)	4.096 (1.128)	0.341 (1.819)	0.265 (1.947)	1.116 (1.911)
	p = 0.000	p = 0.0003	p = 0.852	p = 0.892	p = 0.560
Qualitative	0.813 (1.153)	−0.833 (1.129)	−0.068 (1.809)	−1.961 (1.950)	−1.742 (1.907)
	p = 0.481	p = 0.461	p = 0.971	p = 0.315	p = 0.362
**Controls**					
Gender	6.465 (0.674)	5.056 (0.660)	3.656 (1.090)	2.944 (1.139)	0.849 (1.106)
	p = 0.000	p = 0.000	p = 0.001	p = 0.010	p = 0.443
University	−4.476 (0.700)	−7.953 (0.686)	−4.842 (1.109)	−6.055 (1.187)	−3.591 (1.157)
	p = 0.000	p = 0.000	p = 0.00002	p = 0.00000	p = 0.002
Age 25-44	−2.806 (1.343)	−2.506 (1.315)	−6.606 (2.119)	0.043 (2.229)	−3.912 (2.274)
	p = 0.037	p = 0.057	p = 0.002	p = 0.985	p = 0.086
Age 45-54	−5.492 (1.415)	−4.879 (1.386)	−8.831 (2.289)	−6.152 (2.313)	−6.129 (2.371)
	p = 0.0002	p = 0.0005	p = 0.0002	p = 0.008	p = 0.010
Age 55+	−11.209 (1.286)	−10.879 (1.260)	−13.337 (2.069)	−10.532 (2.110)	−11.661 (2.158)
	p = 0.000	p = 0.000	p = 0.000	p = 0.00000	p = 0.00000
**Interactions**					
Time middle tercile:Vote share	0.911 (1.627)	−1.877 (1.594)	0.818 (2.571)	−0.749 (2.762)	3.443 (2.672)
	p = 0.576	p = 0.240	p = 0.751	p = 0.787	p = 0.198
Time upper tercile:Vote share	0.969 (1.628)	−1.425 (1.595)	2.632 (2.587)	0.408 (2.724)	0.037 (2.686)
	p = 0.552	p = 0.372	p = 0.310	p = 0.882	p = 0.989
Time middle tercile:Probability	−1.933 (1.630)	0.738 (1.596)	1.876 (2.569)	4.104 (2.766)	0.524 (2.680)
	p = 0.236	p = 0.644	p = 0.466	p = 0.139	p = 0.846
Time upper tercile:Probability	−3.596 (1.633)	−2.896 (1.599)	1.905 (2.612)	−0.946 (2.729)	−0.701 (2.678)
	p = 0.028	p = 0.071	p = 0.466	p = 0.729	p = 0.794
Time middle tercile:Qualitative	−0.607 (1.629)	0.898 (1.595)	−1.366 (2.575)	5.529 (2.770)	−0.440 (2.672)
	p = 0.710	p = 0.574	p = 0.596	p = 0.047	p = 0.870
Time upper tercile:Qualitative	−2.409 (1.629)	−0.106 (1.596)	0.373 (2.601)	2.333 (2.729)	0.095 (2.673)
	p = 0.140	p = 0.947	p = 0.886	p = 0.393	p = 0.972
Observations	2,934	2,933	1,000	989	942
R^2	0.110	0.110	0.081	0.095	0.069
Adjusted R^2	0.105	0.105	0.066	0.080	0.053

SM17 Manski Questions

In our pre-registration, we planned to construct 'for each respondent, a Beta distribution representing the probabilities they assign to their vote share expectations' following the approach recommended by (Leemann et al. 2021). However, we found that when attempting to apply this approach, we generated nonsensical distributions. We therefore followed our back-up plan of using the bounds reported by respondents as the range of their subjective distributions.

SM18 Interaction Effect Benjamini Hochberg-Adjusted P-Values

We fit models with interaction effects to assess the heterogeneity of treatment effects by a series of respondent characteristics. To minimise false discovery rates in these analyses, we pre-registered Benjamini-Hochberg adjusted p-values, reported in Tables SM34-SM39. Applying this procedure, the only interaction effects that remain statistically significant capture the variation in the effects of the probabilistic and vote share treatments by levels of support for Jean-Luc Mélenchon (Feeling:Probabilistic and Feeling:Vote Share in Table SM34).

Table SM34: Benjamini-Hochberg adjusted p-values – Candidate Feelings

Effect	Outcome	Raw p-values	Adjusted p-values
Feeling:Probabilistic	Le Pen	0.5161032	0.7741547
Feeling:Qualitative	Le Pen	0.0835895	0.3134605
Feeling:Vote Share	Le Pen	0.0453309	0.2266544
Feeling:Probabilistic	Macron	0.2505741	0.6027594
Feeling:Qualitative	Macron	0.6547810	0.8928832
Feeling:Vote Share	Macron	0.1521740	0.4565221
Feeling:Probabilistic	Mélenchon	0.0007447	0.0085379
Feeling:Qualitative	Mélenchon	0.3053656	0.6027594
Feeling:Vote Share	Mélenchon	0.0011384	0.0085379
Feeling:Probabilistic	Pécresse	0.7470308	0.9337884
Feeling:Qualitative	Pécresse	0.9940118	0.9940118
Feeling:Vote Share	Pécresse	0.4397875	0.7329792
Feeling:Probabilistic	Zemmour	0.9824079	0.9940118
Feeling:Qualitative	Zemmour	0.3214717	0.6027594
Feeling:Vote Share	Zemmour	0.8693964	0.9940118

Table SM35: Benjamini-Hochberg adjusted p-values – Party ID

Effect	Candidate	Raw p-values	Adjusted p-values
Party ID:Probabilistic	Le Pen	0.2269214	0.9053443
Party ID:Qualitative	Le Pen	0.3621377	0.9053443
Party ID:Vote Share	Le Pen	0.2490197	0.9053443
Party ID:Probabilistic	Macron	0.5148625	0.9360987
Party ID:Qualitative	Macron	0.7681808	0.9360987
Party ID:Vote Share	Macron	0.8169732	0.9360987
Party ID:Probabilistic	Mélenchon	0.9360987	0.9360987
Party ID:Qualitative	Mélenchon	0.0119337	0.1790056
Party ID:Vote Share	Mélenchon	0.2074772	0.9053443
Party ID:Probabilistic	Pécresse	0.8669449	0.9360987
Party ID:Qualitative	Pécresse	0.3036180	0.9053443
Party ID:Vote Share	Pécresse	0.9216002	0.9360987
Party ID:Probabilistic	Zemmour	0.7273512	0.9360987
Party ID:Qualitative	Zemmour	0.4678027	0.9360987
Party ID:Vote Share	Zemmour	0.8970738	0.9360987

Table SM36: Benjamini-Hochberg adjusted p-values – Ideological distance

Effect	Candidate	Raw p-values	Adjusted p-values
Distance:Probabilistic	Le Pen	0.2862156	0.7815225
Distance:Qualitative	Le Pen	0.4022293	0.7815225
Distance:Vote Share	Le Pen	0.2204365	0.7815225
Distance:Probabilistic	Macron	0.5484915	0.7846353
Distance:Qualitative	Macron	0.4950476	0.7846353
Distance:Vote Share	Macron	0.0589336	0.4420021
Distance:Probabilistic	Mélenchon	0.2245483	0.7815225
Distance:Qualitative	Mélenchon	0.6027623	0.7846353
Distance:Vote Share	Mélenchon	0.0314971	0.4420021
Distance:Probabilistic	Pécresse	0.6277082	0.7846353
Distance:Qualitative	Pécresse	0.8082252	0.9325676
Distance:Vote Share	Pécresse	0.4168120	0.7815225
Distance:Probabilistic	Zemmour	0.3423421	0.7815225
Distance:Qualitative	Zemmour	0.9372545	0.9372545
Distance:Vote Share	Zemmour	0.8817695	0.9372545

Table SM37: Benjamini-Hochberg adjusted p-values – Political Interest

Effect	Candidate	Raw p-values	Adjusted p-values
Interest:Probabilistic	Le Pen	0.1504834	0.5643126
Interest:Qualitative	Le Pen	0.8070791	0.9312451
Interest:Vote Share	Le Pen	0.3270364	0.6440340
Interest:Probabilistic	Macron	0.0363644	0.2727329
Interest:Qualitative	Macron	0.1317056	0.5643126
Interest:Vote Share	Macron	0.3071929	0.6440340
Interest:Probabilistic	Mélenchon	0.4454337	0.7423896
Interest:Qualitative	Mélenchon	0.2122732	0.6368195
Interest:Vote Share	Mélenchon	0.7042915	0.8803644
Interest:Probabilistic	Pécresse	0.9914047	0.9914047
Interest:Qualitative	Pécresse	0.0295216	0.2727329
Interest:Vote Share	Pécresse	0.6277076	0.8803644
Interest:Probabilistic	Zemmour	0.6515990	0.8803644
Interest:Qualitative	Zemmour	0.3434848	0.6440340
Interest:Vote Share	Zemmour	0.9531246	0.9914047

Table SM38: Benjamini-Hochberg adjusted p-values – Anti-Expert Sentiment

Effect	Candidate	Raw p-values	Adjusted p-values
Anti-Expert:Probabilistic	Le Pen	0.6164091	0.6604384
Anti-Expert:Qualitative	Le Pen	0.1105165	0.5124847
Anti-Expert:Vote Share	Le Pen	0.4245178	0.6077730
Anti-Expert:Probabilistic	Macron	0.4457002	0.6077730
Anti-Expert:Qualitative	Macron	0.5107082	0.6383853
Anti-Expert:Vote Share	Macron	0.2315991	0.5124847
Anti-Expert:Probabilistic	Mélenchon	0.0239097	0.3586448
Anti-Expert:Qualitative	Mélenchon	0.2391595	0.5124847
Anti-Expert:Vote Share	Mélenchon	0.2128225	0.5124847
Anti-Expert:Probabilistic	Pécresse	0.5649060	0.6518146
Anti-Expert:Qualitative	Pécresse	0.3780893	0.6077730
Anti-Expert:Vote Share	Pécresse	0.2187054	0.5124847
Anti-Expert:Probabilistic	Zemmour	0.1867526	0.5124847
Anti-Expert:Qualitative	Zemmour	0.8359908	0.8359908
Anti-Expert:Vote Share	Zemmour	0.3238780	0.6072712

Table SM39: Benjamini-Hochberg adjusted p-values – Over time

Effect	Candidate	Raw p-values	Adjusted p-values
Time Middle Tercile:Probabilistic	Le Pen	0.6436726	0.9843191
Time Middle Tercile:Qualitative	Le Pen	0.5734812	0.9843191
Time Middle Tercile:Vote Share	Le Pen	0.2390401	0.8964004
Time Upper Tercile:Probabilistic	Le Pen	0.0701940	0.7019399
Time Upper Tercile:Qualitative	Le Pen	0.9468260	0.9889585
Time Upper Tercile:Vote Share	Le Pen	0.3715587	0.9843191
Time Middle Tercile:Probabilistic	Macron	0.2355075	0.8964004
Time Middle Tercile:Qualitative	Macron	0.7093893	0.9843191
Time Middle Tercile:Vote Share	Macron	0.5755846	0.9843191
Time Upper Tercile:Probabilistic	Macron	0.0276993	0.6937028
Time Upper Tercile:Qualitative	Macron	0.1393232	0.8359391
Time Upper Tercile:Vote Share	Macron	0.5516100	0.9843191
Time Middle Tercile:Probabilistic	Mélenchon	0.4654468	0.9843191
Time Middle Tercile:Qualitative	Mélenchon	0.5959145	0.9843191
Time Middle Tercile:Vote Share	Mélenchon	0.7503273	0.9843191
Time Upper Tercile:Probabilistic	Mélenchon	0.4658255	0.9843191
Time Upper Tercile:Qualitative	Mélenchon	0.8858872	0.9843191
Time Upper Tercile:Vote Share	Mélenchon	0.3093327	0.9843191
Time Middle Tercile:Probabilistic	Pécresse	0.8451841	0.9843191
Time Middle Tercile:Qualitative	Pécresse	0.8691543	0.9843191
Time Middle Tercile:Vote Share	Pécresse	0.1979004	0.8964004
Time Upper Tercile:Probabilistic	Pécresse	0.7935395	0.9843191
Time Upper Tercile:Qualitative	Pécresse	0.9717731	0.9889585
Time Upper Tercile:Vote Share	Pécresse	0.9889585	0.9889585
Time Middle Tercile:Probabilistic	Zemmour	0.1382856	0.8359391
Time Middle Tercile:Qualitative	Zemmour	0.0462469	0.6937028
Time Middle Tercile:Vote Share	Zemmour	0.7864097	0.9843191
Time Upper Tercile:Probabilistic	Zemmour	0.7288205	0.9843191
Time Upper Tercile:Qualitative	Zemmour	0.3928132	0.9843191
Time Upper Tercile:Vote Share	Zemmour	0.8810225	0.9843191
